# Global trends and stratified analysis of aortic aneurysm mortality: insights from the GBD 2021 study

**DOI:** 10.3389/fcvm.2025.1496166

**Published:** 2025-05-22

**Authors:** Weiguang Yang, Songzhe Wu, Feng Qi, Linlin Zhao, Bo Hai, Haoju Dong, Bufan Zhang, Ruirong Gao, Naishi Wu

**Affiliations:** ^1^ Department of Cardiovascular Surgery, Tianjin Medical University General Hospital, Tianjin, China; ^2^ Department of Cardiovascular Surgery, Chifeng Hospital, Chifeng, Inner Mongolia, China; ^3^ Pediatric Cardiac Surgery, Fuwai Central China Cardiovascular Hospital, Zhengzhou, China

**Keywords:** aortic aneurysm, epidemiology, global burden of disease, sociodemographic index, mortality

## Abstract

**Background:**

Aortic aneurysm (AA) is a serious cardiovascular disease with a high mortality rate. The aim of this study was to provide an up-to-date assessment of global AA mortality rates from 1990 to 2021, stratified by age, sex, and sociodemographic indices (SDI).

**Method:**

This study utilized data from the Global Burden of Disease (GBD) 2021 **analyzed** the global burden of AA from 1990 to 2021. A stratified analysis by sex, age, and region was conducted based on the number of deaths and age-standardized death rates (ASDR). Furthermore, the ASDR trends were evaluated using age-period-cohort models and health inequality analysis methods. Existing data were also used to predict future AA mortality.

**Result:**

In 2021, the global number of deaths due to AA increased to 153,927.20 (95% UI: 138,413.36–165,738.65), while ASDR decreased to 1.86 per 100,000 (95% UI: 1.67–2.00). The estimated annual percentage change in ASDR was −0.81 (95% CI: −0.95 to −0.66), and the average annual percentage change was −0.99 (95% CI: −1.07 to −0.90). Significant regional differences were observed, with high SDI regions experiencing a notable decrease in mortality rates, while low and low-middle SDI regions saw a substantial increase in deaths and an upward trend in mortality rates. The annual percentage change in mortality rates for those aged 55–59 years and older was positive, identifying this age group as a significant risk factor for increased AA-related mortality. At the national level, the three countries with the highest ASDR were Armenia, Montenegro, and Nauru, while the three countries with the lowest ASDR were Saudi Arabia, Tajikistan, and Afghanistan. Predictive analyses suggest that although the number of AA-related deaths is expected to continue rising, mortality rates are projected to decline.

**Conclusion:**

While the global number of deaths from AA is anticipated to keep increasing, the ASDR is generally declining due to advances in medical care and improvements in early intervention. However, there are significant differences between countries and SDI regions. In low and low-middle SDI regions, the ASDR continues to rise, whereas in high and high-middle SDI regions, the ASDR is steadily decreasing but still remains higher than in lower SDI regions. Therefore, early screening and effective management of high-risk groups are crucial on a global scale.

## 1 Introduction

Aortic aneurysm (AA) is a disease involving the aortic wall, characterized by abnormal expansion of the aorta in some parts, usually the diameter of the aorta expands by more than 50% of the normal value. According to the location of occurrence, AA can be divided into thoracic aortic aneurysm and abdominal aortic aneurysm. Abdominal aortic aneurysm is a common form, especially in the elderly. Thoracic aortic aneurysm is more prone to dissection and rupture due to the increased arterial pressure (1–3). AA have no obvious symptoms in the early stages. As the aneurysm expands, the risk of rupture or dissection increases significantly (4–6). According to 2019 statistics, the disease burden of AA in China increased significantly from 1990 to 2019 (7, 8), In a survey of the Washington, U.S., population, of the 1,014,039 deaths that occurred in Washington state during the study period, 4,438 (0.4%) listed AA as an underlying or related cause of death. Global statistics suggest that AA cause hundreds of thousands of deaths. People die, and its morbidity and mortality rates have increased significantly among the population due to global aging (1, 9, 10). Despite great advances in medical intervention and surgical techniques, the global burden of AA remains significant.

The Global Burden of Disease (GBD) database is one of the global health databases, covering the global, regional, and national levels of disease and risk factor burden research from 1990 to 2021. GBD provides detailed assessment tools for the incidence, mortality, and disability rates of a variety of diseases. For AA, the GBD database provides key global and regional epidemiological data, which can track the incidence, mortality, and other trends of the disease in different countries and populations. It is of great significance to understand the global health burden of AA, identify high-risk populations, and evaluate the effectiveness of intervention measures.

This study systematically analyzed AA, a major disease, using data from the GBD study (11), focus is on the global disease burden between 1990 and 2021. The study not only counts core indicators such as the number of deaths and age-standardized death rates (ASDR), but also (12),We evaluated the mortality trends of AA among different genders, age groups and sociodemographic indices (SDI) regions around the world, and also revealed significant differences between different groups through stratified analysis.

## 2 Material and method

### 2.1 Data source

The methodological details of the Global Burden of Disease, Injuries, and Risk Factors Study GBD 2021 have been previously published (7, 13), The data for this study were derived from the GBD 2021 database, which provides comprehensive information on the burden of AA, including indicators such as deaths, ASDR, average annual percentage change (AAPC), and estimated annual percentage change (EAPC). Data were collected from 204 countries and regions and stratified by sex, age group, and SDI.

### 2.2 Research design and analytical framework

This retrospective observational study evaluated global trends in AA burden by analyzing GBD historical data from 1990 to 2021. Stratified analyses were conducted across sex, age groups, and SDI-based regions to explore mortality trends in diverse populations.

### 2.3 Analytical indicators

Deaths: refers to the total number of deaths caused by AA between 1990 and 2021. ASDR: Age-standardized mortality rate per 100,000 population, adjusted for population age structure to enable cross-temporal and cross-regional comparisons; EAPC: used to evaluate the annual average change of ASDR, the calculation formula is the linear regression coefficient of the natural logarithm of ASDR over time, used to quantify the changing trend of AA mortality rate (14), AAPC: The annualized rate of change in different time periods was estimated using a Joinpoint regression model (15, 16), to evaluate the long-term trend of aortic aneurysm ASDR.

### 2.4 Statistical models and methods

This study used the following statistical models and methods: Age Period Cohort-Intrinsic Estimator (APC-IE) Model: This model is used to evaluate the changing trend of mortality caused by AA in different birth cohorts (such as people born in different years) at a specific age. By dividing the population by age, period and birth cohort, the APC model can identify the changes in risk at different time points and in different age groups, thereby more accurately evaluating the impact of demographic factors on mortality (17). Health inequality analysis: In order to assess the health inequality of AA disease burden worldwide, the study introduced the Concentration Index to quantify the inequality in the distribution of health resources between regions (18, 19). By analyzing mortality rates in different SDI regions, we identified health inequalities between high-income and low-income areas and further explored the impact of epidemiological changes on health inequalities, Joinpoint regression analysis: This method is used to identify the trend of ASDR over time and find possible change points (20, 21). This study used a multi-segment linear regression model to find the turning points of ASDR changes and calculated the annual change rate for each period to better understand the changes in mortality rates in different periods.

### 2.5 Future trend prediction

Using the Nordpred model, we projected AA-related deaths and mortality rates from 2022 to 2046. Stratified projections by sex and age were generated to assess future burden dynamics.

### 2.6 Data processing and statistical analysis tools

Data processing and analysis were performed using the R software (version 4.3.1) and Stata software provided in the GBD project. The pre-set model in the GBD analysis framework was used for the estimation of ASDR and the calculation of EAPC, and stratified analysis was added to improve the accuracy of the results. For health inequality analysis and future predictions, the study used standard statistical methods in health economics and combined multiple models for comparative verification.

## 3 Result

### 3.1 ASDR for AA and its average annual change in the world and across SDI regions (1990–2021)

**Figure 1:**
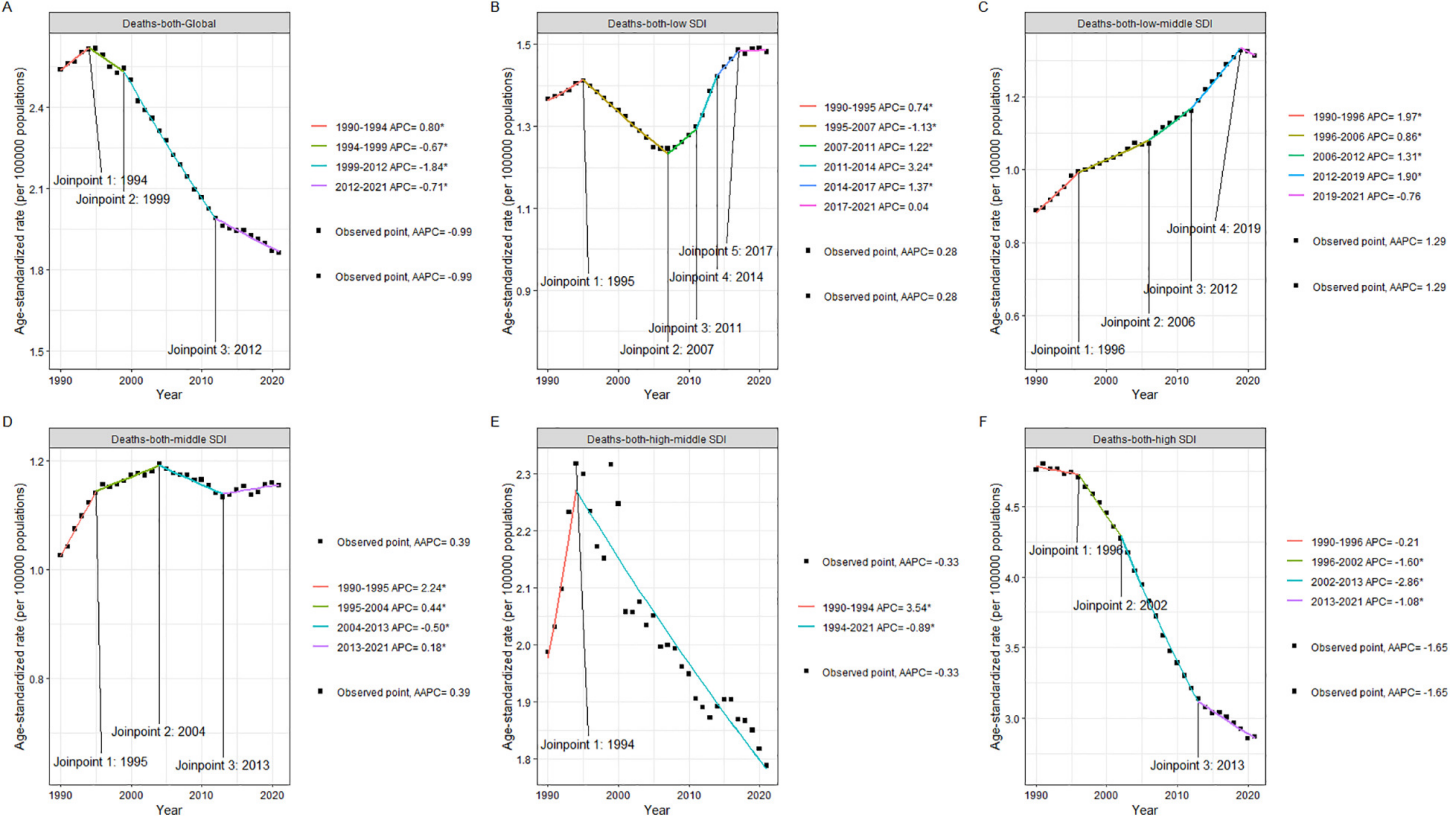
Joinpoint plot of ASDR and its mean annual change in AA by region of the global and different SDI from 1990 to 2021. **(A)** Global. **(B)** Low SDI. **(C)** Low-middle SDI. **(D)** Middle SDI. **(E)** High-middle SDI. **(F)** High SDI.

As can be seen from Table 1, taking the world as an example, in 1990, the number of deaths caused by AA was 88352.86 (95% UI: 83,090.19–93,491.94), and the ASDR was 2.54 (95% UI: 2.35–2.69); by 2021, the number of deaths increased to 153,927.20 (95% UI: 138,413.36–165,738.65), and the ASDR dropped to 1.86 (95% UI: 1.67–2.00); between 1990 and 2021, the average annual change in ASDR EAPC was −0.81 (95% CI: −0.95 to −0.66), and AAPC was −0.99 (95% CI: −1.07 to −0.90). It can be seen from Figure 1 that the ASDR Join point of AA has changed three times globally from 1990 to 2021, in 1994, 1999 and 2012 respectively; among them, the decline from 1990 to 2012 was the most significant, with an annual change rate of −1.84.

**Table 1:** Disease burden of AA in the world and different SDI, 1990–2021.

Location	1990 Number	1990 ASDR (95% UI) (per 100,000)	2021 Number	2021 ASDR (95% UI) (per 100,000)	1990–2021 EAPC (95% CI)	1990–2021 AAPC (95% CI)
Global	88,352.86 (83,090.19–93,491.94)	2.54 (2.35–2.69)	153,927.20 (138,413.36–165,738.65)	1.86 (1.67–2.00)	−0.81(−0.95 to −0.66)	−0.99(−1.07 to −0.90)
Low SDI	2,556.85 (1,567.67–4,436.97)	1.37 (0.83–2.37)	6371.33 (3,931.56–10,433.65)	1.48 (0.91–2.44)	0.14 (0.02–0.27)	0.28 (0.13–0.43)
Low-middle SDI	4,608.30 (3,663.78–6,271.72)	0.89 (0.71–1.20)	16,808.25 (13,956.42–22,468.09)	1.31 (1.09–1.76)	1.29 (1.26–1.33)	1.29 (1.17–1.40)
Middle SDI	8,804.38 (8,109.98–9,844.14)	1.03 (0.94–1.14)	28,528.43 (25,797.27–30,958.58)	1.15 (1.04–1.25)	0.63 (0.48–0.77)	0.39 (0.30–0.47)
High-middle SDI	18,320.67 (17,507.75–19,196.64)	1.98 (1.88–2.08)	34,826.60 (32,309.33–37,274.13)	1.79 (1.66–1.92)	−0.01(−0.21 to 0.20)	−0.33(−0.61 to −0.06)
High SDI	53,929.43 (50,581.52–55,553.20)	4.76 (4.46–4.91)	67,201.85 (57,735.40–72,287.48)	2.87 (2.51–3.06)	−1.37(−1.56 to −1.18)	−1.65(−1.74 to −1.56)

SDI, sociodemographic index; ASDR, age- standardized death rate; 95%UI, 95% uncertainty intervals; EAPC, estimated annual percentage change; AAPC, average annual percentage change; 95%CI, 95% confidence interval.

In Low SDI areas, the number of deaths caused by AA in 1990 was 2,556.85 (95%UI: 1,567.67–4,436.97), and the ASDR was 1.37 (95%UI: 0.83–2.37); the number of deaths caused by AA in 2021 was 6,371.33 (95%UI: 3,931.56–10,433.65), and the ASDR was 1.48 (95%UI: 0.91–2.44); from 1990 to 2021, the number of deaths increased by 2.5 times (from 2,556.85 to 6,371.33); the ASDR increased slightly by 0.11 (from 1.37 to 1.48), an increase of about 8.0; between 1990 and 2021, the EAPC was 0.14 (95%CI: 0.02–0.27), although this growth exists, the amplitude is relatively small; AAPC is 0.28 (95%CI: 0.13–0.43), indicating that although the mortality rate in this area has increased, the overall trend is relatively slow. As can be seen from Figure 1, the ASDR Joinpoint of AA in the Low SDI area has changed five times from 1990 to 2021, in 1995, 2007, 2011, 2014, and 2017 respectively; among them, only from 1995 to 2017 did the ASDR show a downward trend, and the rest of the years showed an upward trend.

In the Low-middle SDI area, the number of deaths caused by AA in 1990 was 4,608.30 (95% CI: 3,663.78–6,271.72), and the ASDR was 0.89 (95% UI: 0.71–1.20); the number of deaths caused by AA in 2021 was 16,808.25 (95% UI: 13,956.42–22,468.09), and the ASDR was 1.31 (95% UI: 1.09–1.76); it increased by about 3.6 times from 4,608 in 1990 to 16,808 in 2021; the ASDR increased from 0.89 in 1990 to 1.31 in 2021, showing that the mortality rate in the area has increased during this period. From 1990 to 2021, the EAPC in the Low-middle SDI area was 1.29 (95% CI: 1.26–1.33); the AAPC was 1.29 (95% CI: 1.17–1.40), indicating that the mortality rate in the area increased at an annual rate of about 1.29 throughout the period, and the trend was relatively stable and obvious. As can be seen from Figure 1, the ASDR Joinpoint of AA in the Low-middle SDI area changed four times from 1990 to 2021, in 1996, 2006, 2012, and 2019, respectively; among them, the ASDR showed a downward trend only from 2019 to 2021, and an upward trend was shown in the remaining years. The upward trend was most obvious during the period from 1990 to 1996, with an annual change rate of 1.97.

In the Middle SDI area, the number of deaths due to AA in 1990 was 8,804.38 (95%UI: 8,109.98–9,844.14), and the ASDR: 1.03 (95%UI: 0.94–1.14); the number of deaths due to AA in 2021 was 28,528.43 (95%UI: 25,797.27–30,958.58), and the ASDR: 1.15 (95%UI: 1.04–1.25); it increased from 8,804 in 1990 to 28,528 in 2021, an increase of about 3.24 times. This shows that the number of AA-related deaths in the Middle SDI region has increased significantly over the past 30 years; despite the significant increase in the number of deaths, the ASDR increased from 1.03 to 1.15, a relatively small increase, indicating that even if more people died, the increase in mortality was not significant considering the age structure of the population; from 1990 to 2021, the EAPC in the Middle SDI region was 0.63 (95% CI: 0.48–0.77); the AAPC was 0.39% (95% CI: 0.30–0.47). As can be seen from Figure 1, the ASDR Joinpoint of AA in the Middle SDI region changed three times from 1990 to 2021, in 1995, 2004, and 2013, respectively. Except for the downward trend from 2004 to 2013, the rest showed an upward trend.

For the High-Middle SDI area, the number of deaths due to AA in 1990 was 18,320.67 (95%UI: 17,507.75–19,196.64), and the ASDR was 1.98 (95%UI: 1.88–2.08); the number of deaths due to AA in 2021 was 34,826.60 (95%UI: 32,309.33–37,274.13), and the ASDR was 1.79 (95%UI: 1.66–1.92); it increased from 18,320 in 1990 to 34,826 in 2021, an increase of nearly 1.9 times; despite the sharp increase in the number of deaths, the ASDR decreased from 1.98 in 1990 to 1.79 in 2021, showing a slight decrease in the mortality rate in the region. This means that although the number of deaths is increasing, the ASDR has actually decreased slightly considering changes in the population structure (such as aging); from 1990 to 2021, the ASDR in the High-Middle SDI region remained basically stable, with an EAPC of −0.01 (95%CI: −0.21–0.20), close to 0, and the AAPC showed that the average annual change in ASDR was −0.33 (95%CI: −0.61 to −0.06), indicating that during these 30 years, the mortality rate in the region has generally decreased slightly. As can be seen from Figure 1, in the High-Middle SDI region, the Joinpoint of ASDR appeared in 1994, and there was a clear downward trend after 1994, with an annual rate of change of −0.89.

As for the High SDI areas, the number of deaths due to AA in 1990 was 53,929.43 (95%UI: 50,581.52–55,553.20), and the ASDR was 4.76 (95%UI: 4.46–4.91). The number of deaths due to AA in 2021 was 67,201.85 (95%UI: 57,735.40–72,287.48), and the ASDR was 2.87 (95%UI: 2.51–3.06); from 53,929 in 1990 to 67,201 in 2021, the number of deaths increased by about 25%. This increase may be related to the aging of the population and the improvement of the early diagnosis rate of aortic aneurysms; despite the increase in the number of deaths, the ASDR decreased from 4.76 in 1990 to 2.87 in 2021, showing a significant decline in AA-related mortality, indicating that medical progress, early screening and effective treatment strategies in the region have played a significant role in reducing mortality. From 1990 to 2021, the EAPC of ASDR in the High SDI region was −1.37 (95 CI: −1.56 to −1.18). This is a significant downward trend, indicating that the region has made effective progress in the prevention, management and treatment of AA; AAPC showed a significant decline in mortality over the entire period of −1.65 (95% confidence interval: −1.74 to −1.56), further supporting the trend of a significant decline in mortality over the past 30 years. This decline rate is higher than that of other SDI categories, indicating that high SDI regions have obvious advantages in health management and medical technology. As can be seen from Figure 1, the ASDR of High SDI shows an obvious downward trend as a whole. From 1990 to 2021, there are three Joinpoints, namely 1996, 2002, and 2013. Among them, the decline from 2002 to 2013 is the most obvious, with an annual change rate of −2.68.

### 3.2 Age-standardized rates (ASR) of AA across geographic regions, age groups, and sex stratifications in 2021

**Figure 2:**
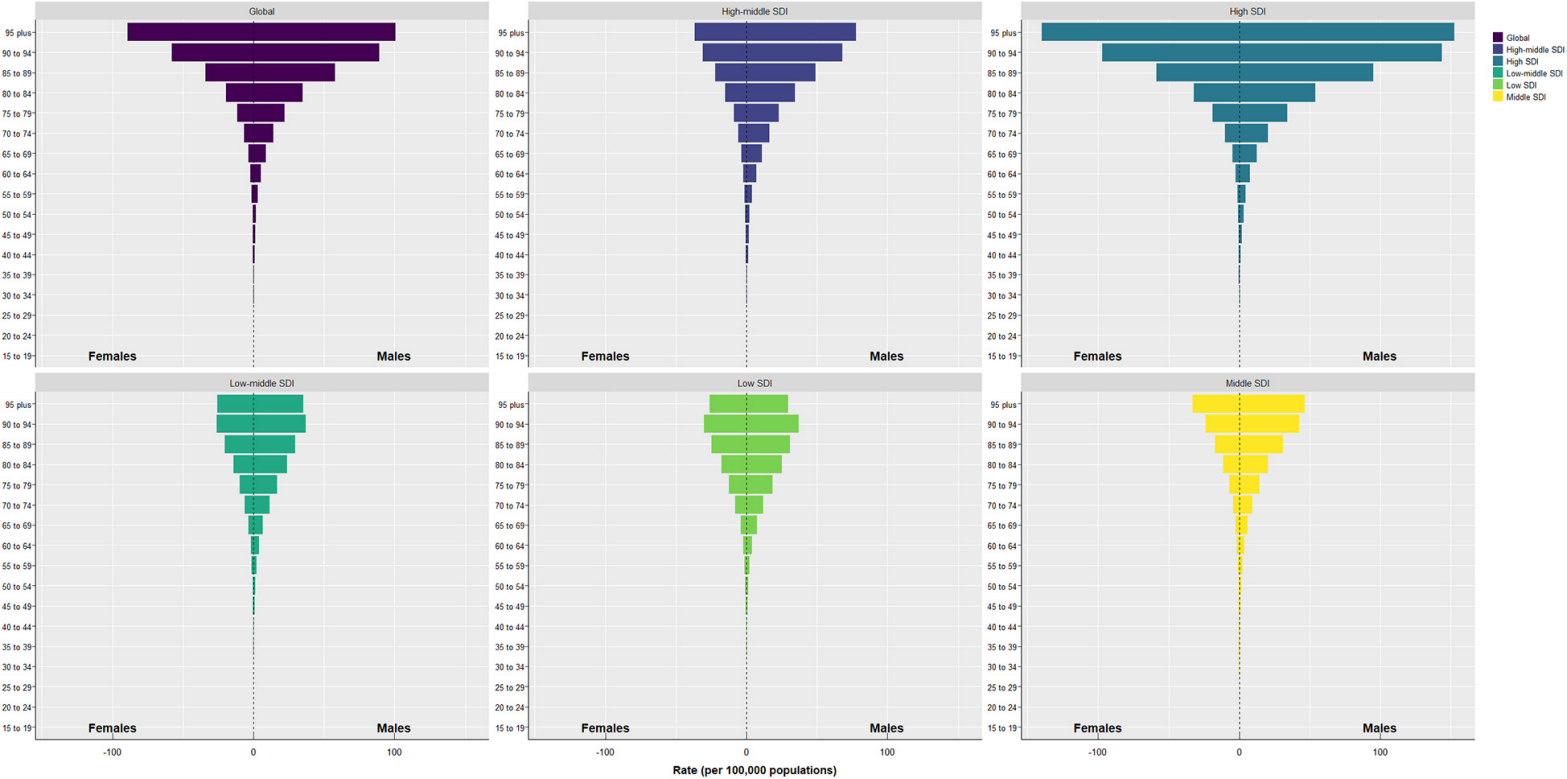
Pyramid chart of mortality rate caused by AA in different regions, different ages and different genders from 1990 to 2021.

Compare the mortality rates of different regions in the world according to SDI classification. These classifications include global and 5 SDI regions, from 15 to 19 years old to over 95 years old, and lists the mortality rates of different genders. According to Figure 2, the older the age, the higher the mortality rate caused by AA. In all regions of the world, the mortality rate of male AA is generally higher than that of female, and the mortality rate of AA in High-SDI regions is higher than that in other regions (Supplementary Table S1). This may be related to the aging of the population in high-level regions and the improvement of detection and diagnosis.

### 3.3 Analysis of the number of deaths and influencing factors at different SDI levels in the world from 1990 to 2021

**Figure 3:**
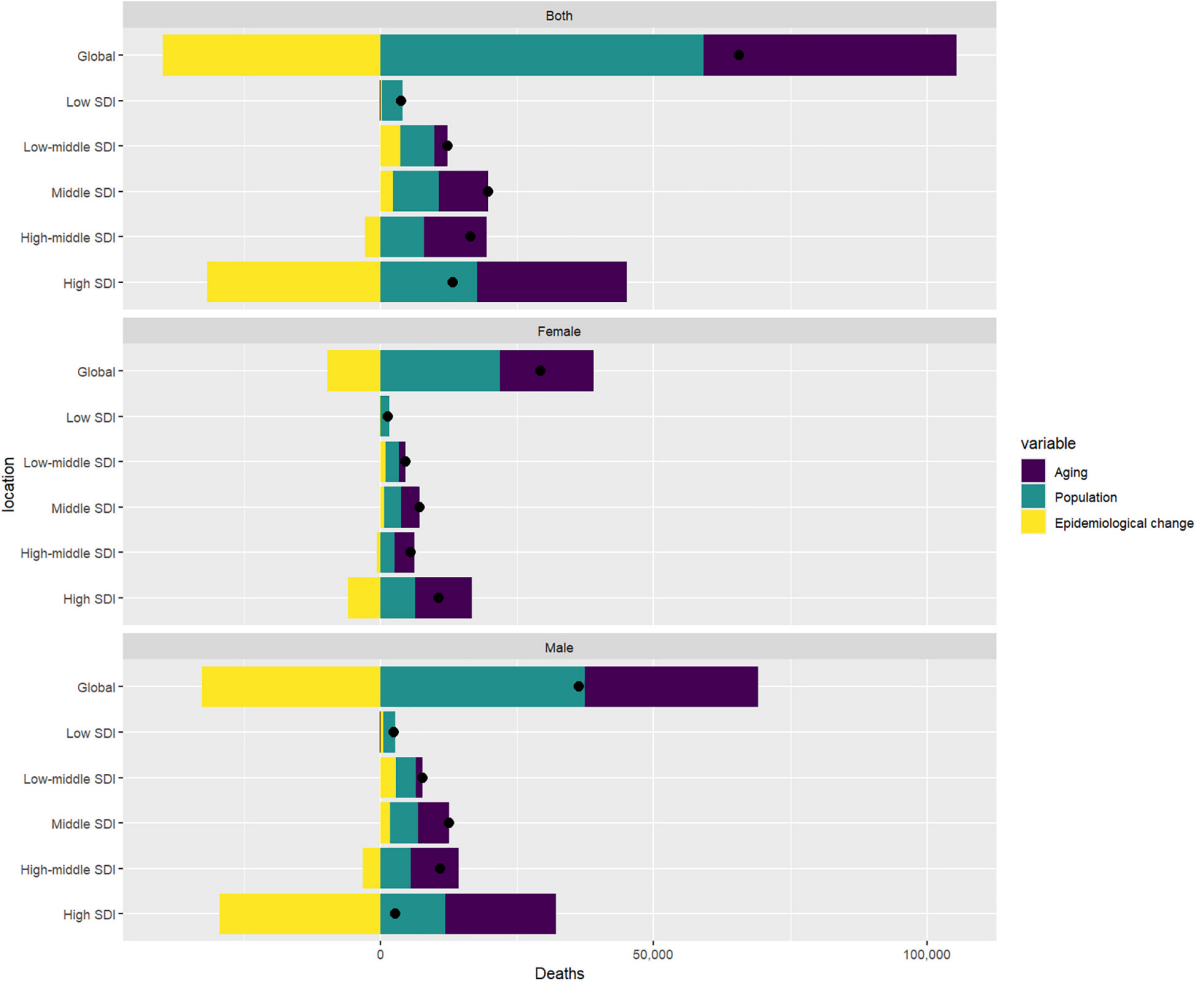
Changes in AA-related mortality rates that occurred early on a global scale from 1990 to 2021, as determined by population levels of population growth, ageing, and epidemiological changes, through the SDI quintile, black dots represent the total value of the changes contributed by all 3 components. For each component, the magnitude of the positive value indicates a corresponding increase in mortality from AA; The magnitude of a negative value indicates a corresponding reduction in mortality from AA attributed to the relevant component.

Table 2 combined with Figure 3 shows that the number of deaths worldwide has increased significantly from 1990 to 2021. Among the five regions divided by SDI, High SDI has the largest increase. Aging and population growth account for 70.74% and 90.14% of the increase in global deaths, respectively. On the contrary, the impact of epidemiological changes on global deaths is negative (−60.88%). In terms of gender, population growth accounts for the largest number of deaths caused by AA in men and women, 102.93% and 74.63% respectively. For Low SDI and Low-Middle SDI regions, population growth has the greatest impact on the growth of deaths, 96.20% and 50.54% respectively. For Middle SDI, High-Middle SDI and High SDI regions, aging has the greatest impact on the growth of deaths, 46.05%, 69.67% and 206.34% respectively. Combined with Figure 3, it can be seen that the main threat and burden of AA in the High SDI region are the aging of the population, while for men around the world, the main threat and burden of AA are population growth. In terms of epidemiological changes, with the Middle region as the boundary, the epidemiological changes in the Low SDI, Low-Middle SDI and Middle SDI regions are all positive, while the epidemiological changes in the High-Middle SDI and High SDI regions are all negative, with the High SDI region being the most significant, reaching −239.50%, indicating that the death rate caused by AA diseases in the High SDI region has been significantly reduced, which is consistent with the research of Krafcik et al. (22) up to 2019. From 1990 to 2021, the medical level and treatment methods in this region have shown significant progress.

**Table 2:** Analysis of deaths and influencing factors at different SDI levels in the world from 1990 to 2021.

Location	Sex	Overall difference	Ageing	Population	Epidemiological change
Global	Male	36,306.91	31,697.05 (87.30%)	37,371.49 (102.93%)	−32,761.63(−90.24%)
	Female	29,267.43	17,218.62 (58.83%)	21,842.55 (74.63%)	−9,793.74(−33.46%)
	Both	65,574.34	46,385.38 (70.74%)	59108.61 (90.14%)	−39,919.65(−60.88%)
Low SDI	Male	2,439.87	−173.48(−7.11%)	2,102.17 (86.16%)	511.18 (20.95%)
	Female	1,374.61	2.33 (0.17%)	1,565.42 (113.88%)	−193.13(−14.05%)
	Both	3,814.48	−147.51(−3.87%)	3,669.69 (96.20%)	292.31 (7.66%)
Low-middle SDI	Male	7,679.68	1,174.12 (15.29%)	3,657.29 (47.62%)	2,848.27 (37.09%)
	Female	4,520.28	1,149.93 (25.44%)	2,485.88 (54.99%)	884.46 (19.57%)
	Both	12,199.95	2,413.57 (19.78%)	6,166.46 (50.54%)	3,619.93 (29.67%)
Middle SDI	Male	12,581.9	5,696.69 (45.28%)	5,177.17 (41.15%)	1,708.05 (13.58%)
	Female	7,142.15	3,436.69 (48.12%)	3,081.94 (43.15%)	623.52 (8.73%)
	Both	19,724.05	9,082.3 (46.05%)	8,325.29 (42.21%)	2,316.46 (11.74%)
High-middle SDI	Male	10,938.98	8,714.93 (79.67%)	5,554.65 (50.78%)	−3,330.6(−30.45%)
	Female	5,566.95	3,729.5 (66.99%)	2,500.56 (44.92%)	−663.11(−11.91%)
	Both	16,505.93	11,499.35 (69.67%)	7,938.64 (48.10%)	−2,932.06(−17.76%)
High SDI	Male	2,632.91	20,302.06 (771.09%)	11,841.51 (449.75%)	−29,510.66(−1120.84%)
	Female	10,639.51	10,300.97 (96.82%)	6,348.24 (59.67%)	−6,009.7(−56.48%)
	Both	13,272.42	27,386.3 (206.34%)	17,673.59(133.16%)	−31,787.47(−239.50%)

SDI, sociodemographic index.

### 3.4 Age-period-cohort model and mortality coefficient for deaths

**Figure 4:**
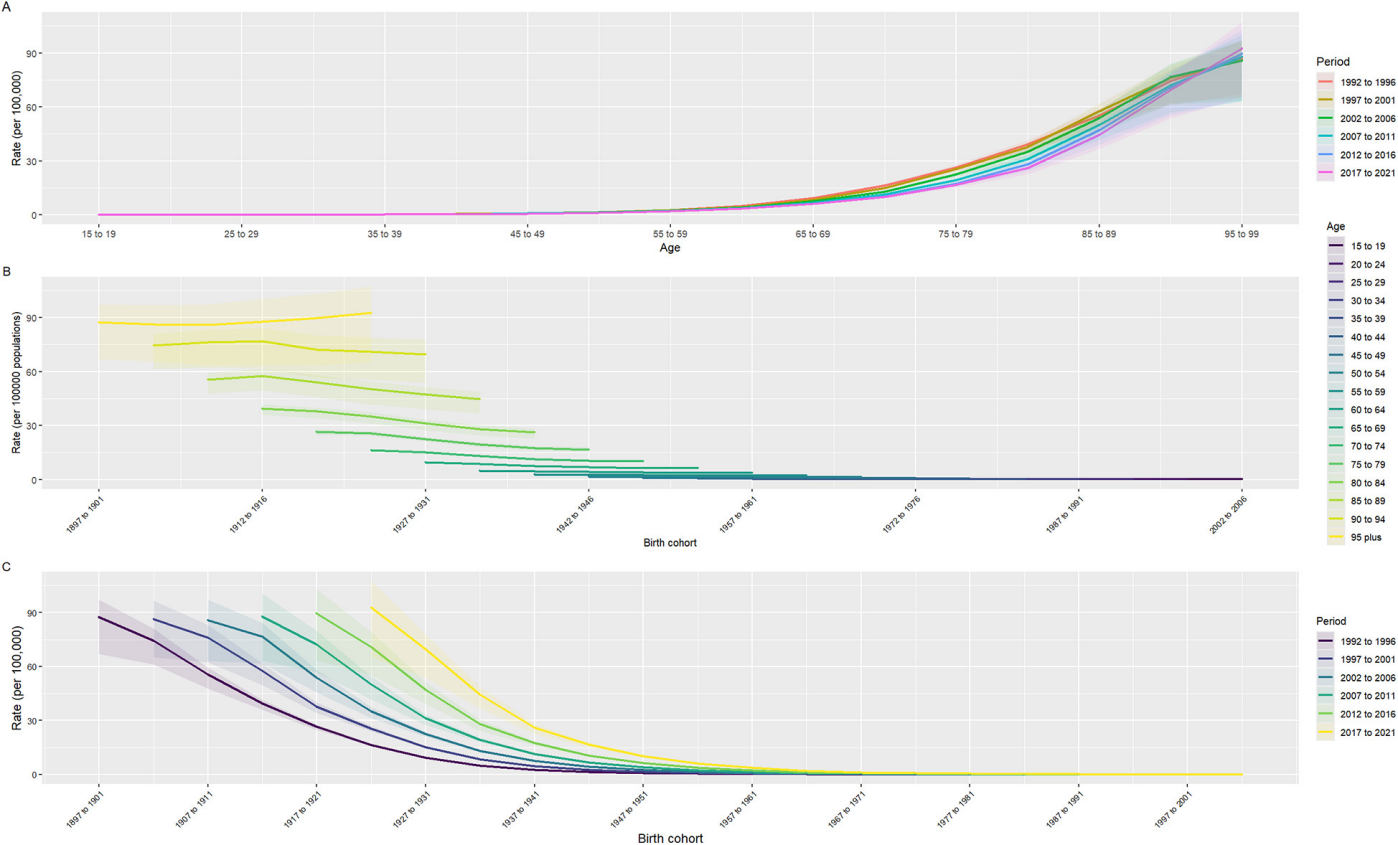
Age-period-cohort model. **(A)** Age is divided by a 5-year-old period, and the curve represents the change in mortality with the change of age period; **(B)** The relationship between birth cohorts and death rates, where different colors represent different age groups; **(C)** The relationship between birth cohort and mortality rate, where different colors represent different period.

**Figure 5:**
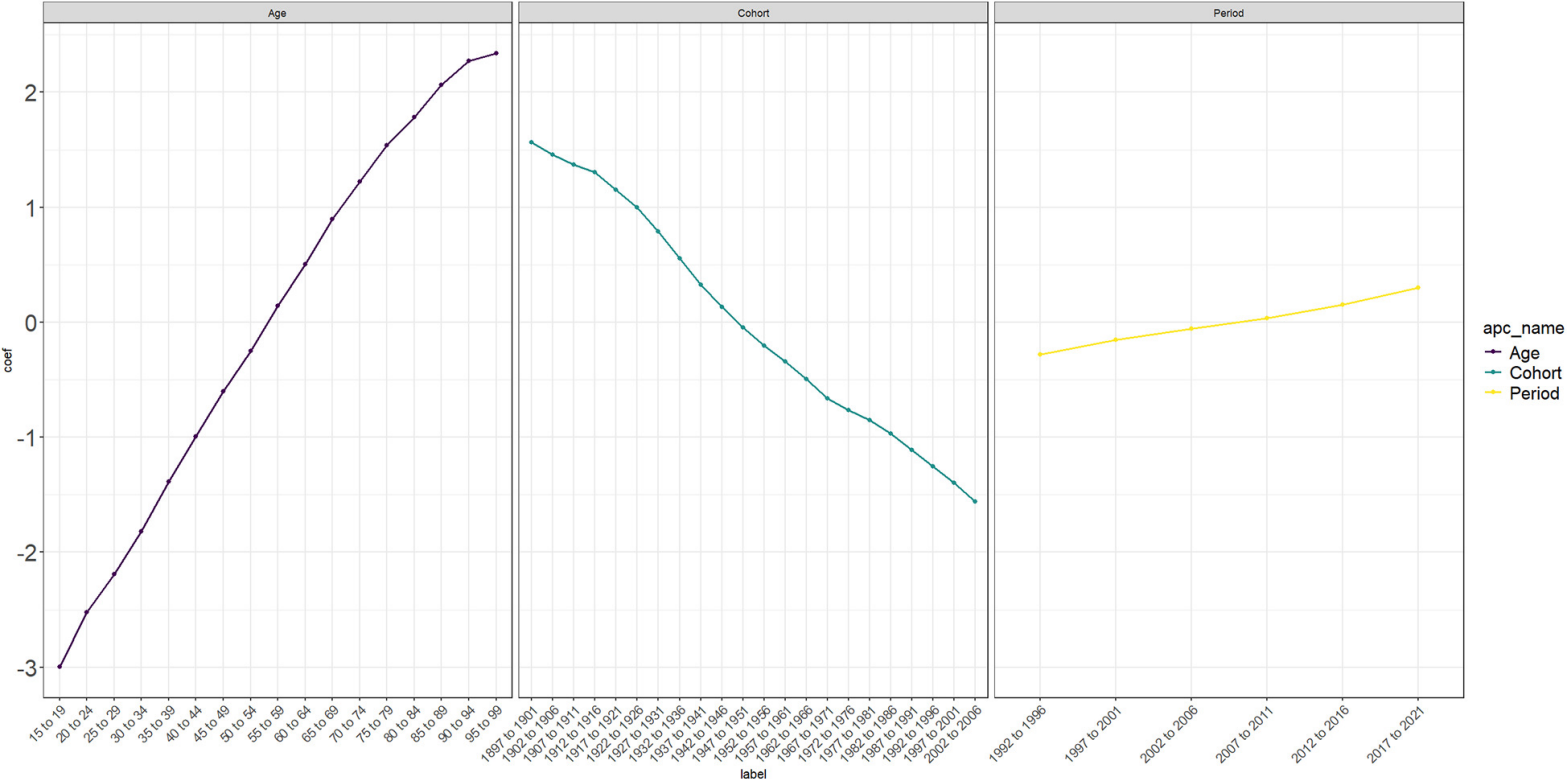
Age-period-cohort model coefficient. The changes in Death rate were observed by different ages, birth cohorts, and period.

By using the Deaths indicator of AA, an age-period-cohort model is established, as shown in Figure 4. As shown in Figure A, the age of 15–99 is divided into a 5-year cycles, and the lines of different colors represent different years. It can be seen that the mortality rate of AA increases with age. For Figure B, the number of people born in 1912–1916 and those born in 1927 and 1931 who died from AA is relatively large, and most of them are between 80 and 94 years old. It can be seen from Figure C in Figure 4. When the birth cohort is divided into 5-year intervals, the relationship between different year periods and different birth cohorts can be seen. For example, most of the people who died from AA from 2017 to 2021 were born between 1922 and 1926; while the number of people who died from AA from 1997 to 2021 was almost zero. In combination with Figure 5, it can be roughly seen that the coefficient of APC exceeds 0 when the age is 55–59 years old, which means that once the age exceeds 55–59 years old, the Death APC becomes a positive number, proving that the annual ASDR will double (Supplementary Table S2). The APC coefficient of people born between 1947 and 1951 is about 0, and the Death APC coefficient of people born before that is positive. Between 2007 and 2011, the APC coefficient was positive, which may be related to population growth and the improvement of diagnostic technology.

### 3.5 Analysis of health inequality in the deaths index between different SDIs from 1990 to 2021

**Figure 6:**
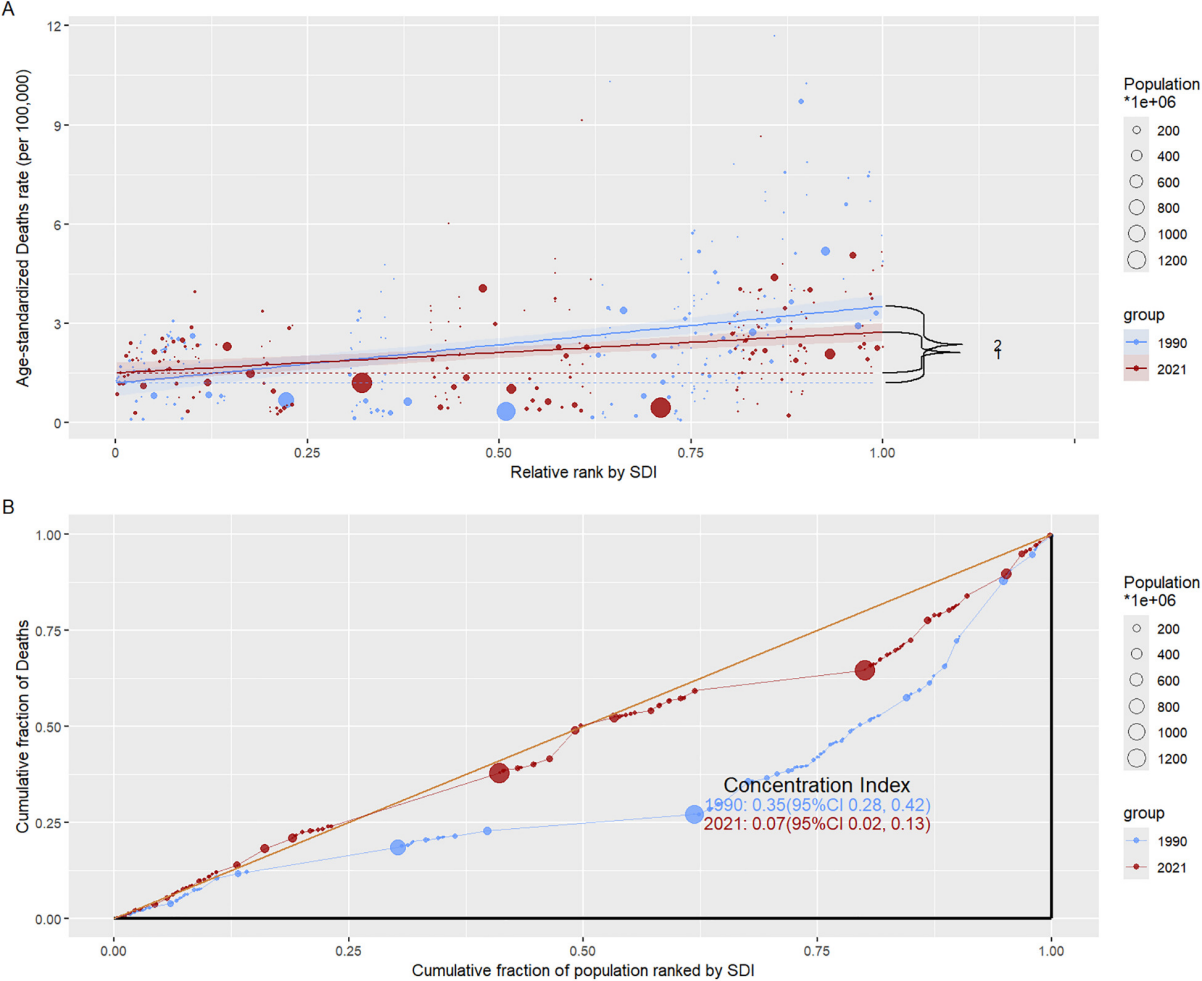
Analysis of mortality inequality in different SDI regions. **(A)** Relationship between relative rank and ASDR for SDI regions in 1990 and 2021; **(B)** The relationship between the cumulative score of the overall ranking by SDI and the cumulative score of death in 1990 and 2021, where Concentration Index stands for a relative measure of health inequality and reflects the distribution of health indicators in socioeconomic status. A value close to 0 indicates that the inequality is smaller, a positive value indicates that the given group is in good health, and a negative value indicates that the poor group is in good health.

According to Figure A in Figure 6, the analysis of health inequality in mortality rates in different SDI regions in 1990 and 2021 shows that the data curves in 1990 and 2021 begin to become discrete at the SDI relative ranking of 0.225, and after this cutoff point, the data curve in 1990 is always above that in 2021. This proves that in relatively higher SDI regions, the mortality rate caused by AA in 2021 has decreased compared with 1990, the mortality rate caused by AA in 1990 is higher than that in 2021, and the mortality rate in different SDI groups in 2021 is generally lower than that in 1990, indicating that with the development of economic and medical technology, the mortality rate of AA has improved worldwide. As shown in Figure 6B, in 1990 and 2021, the Concentration Index of Deaths was 0.35 (95%CI: 0.28, 0.42) and 0.07 (95%CI: 0.02, 0.13), respectively. Its Concentration Index has decreased, indicating that health inequality has weakened. In general, between 1990 and 2021, with the development of social and medical technology, health inequality has improved, and the mortality rate in low SDI areas has decreased.

### 3.6 Correlation between ASDR and SDI at national and regional levels

**Figure 7:**
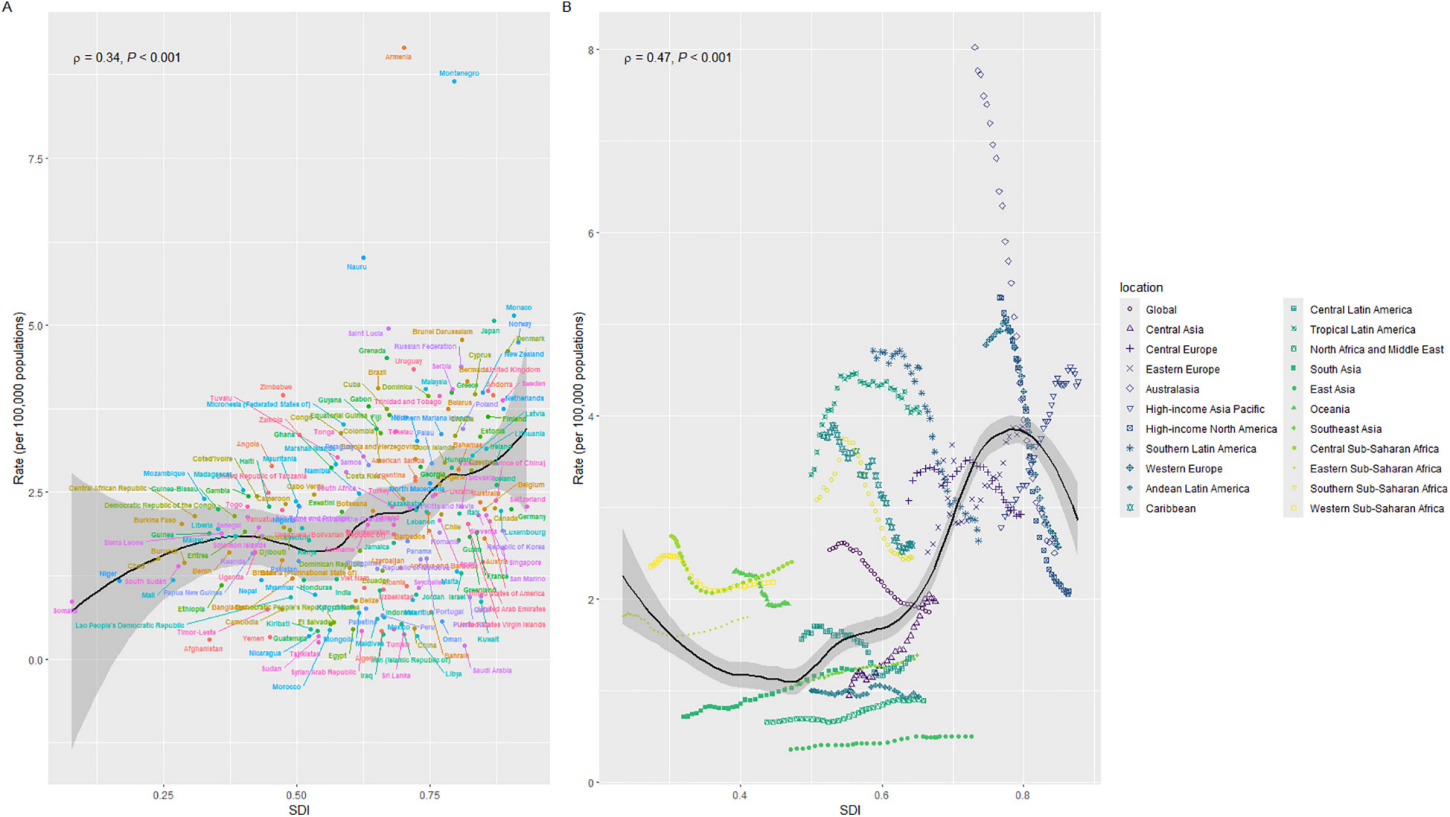
Correlation of mortality rates with SDI regions. **(A)** Correlation between individual countries and mortality rates in different SDI regions. **(B)** Correlation between different SDI regions and mortality by geographic location; *ρ* represents the correlation of Spearman's analysis, indicating that SDI regions are positively correlated with mortality.

**Figure 8:**
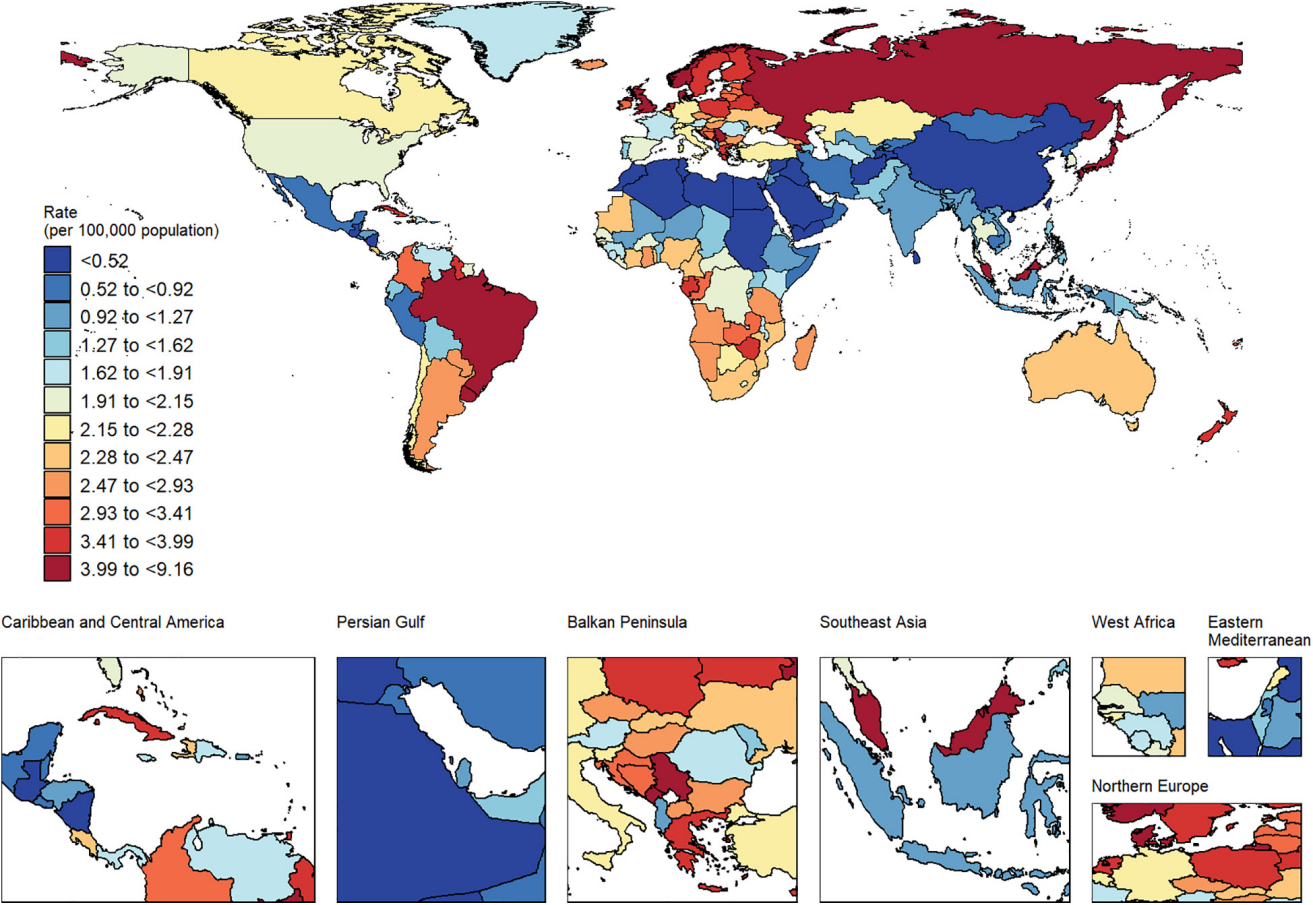
ASDR caused by AA in countries around the world in 2021.

Figure 7A shows a positive correlation between ASDR and SDI at the national level in 2021 (*ρ* = 0.34, *P* < 0.001). Similarly, Figure 7B demonstrates a positive correlation between ASDR and SDI at the regional level (*ρ* = 0.47, *P* < 0.001). In these figures, the curves represent the trends of change, while the shaded areas indicate the range of variability. Combined with Figure 8, these visualizations provide a straightforward way to observe the ASDR attributable to disease burden across different countries.

### 3.7 Projection of mortality rates and cases attributable to AA through 2046

**Figure 9:**
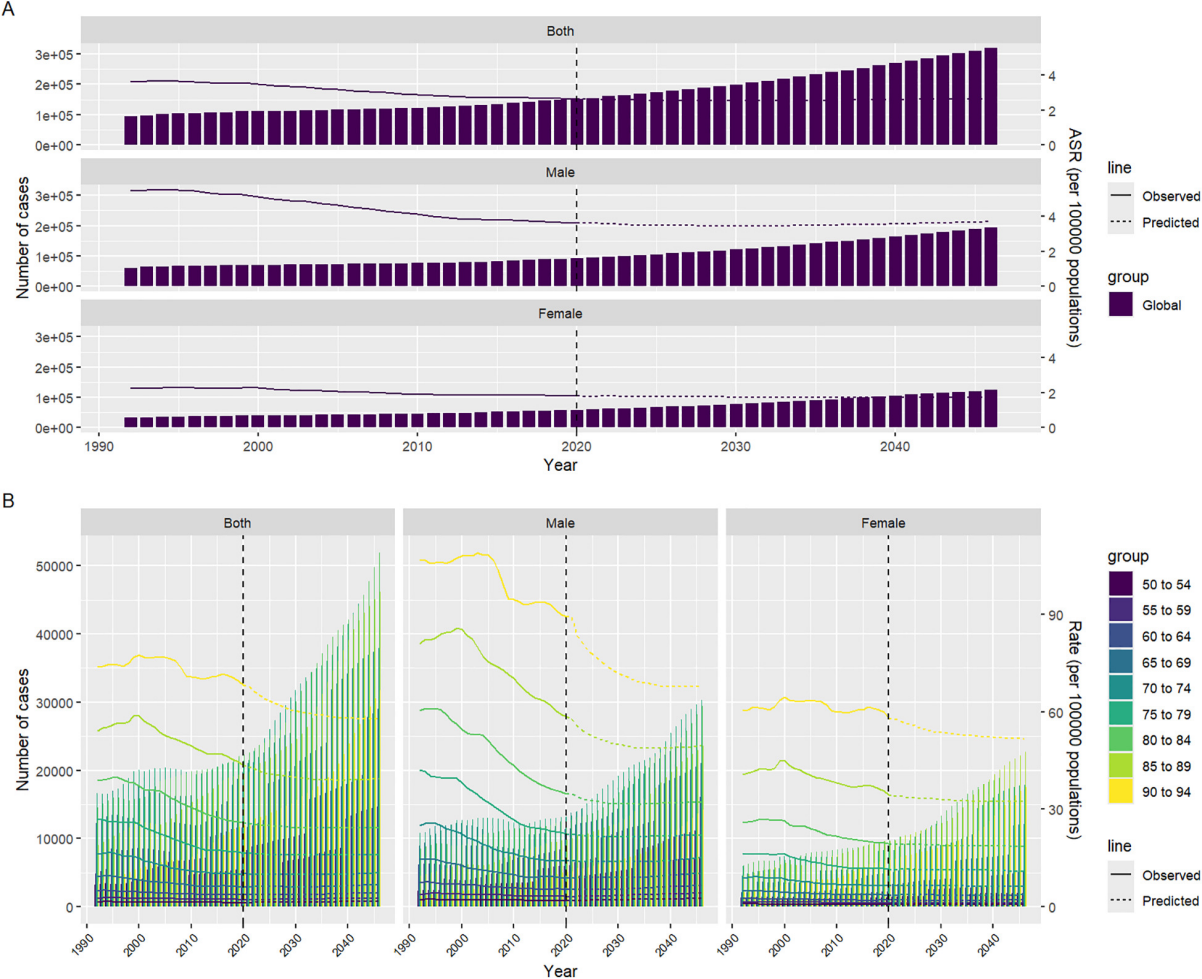
Predicts the development of ASDR due to AA from 2022 to 2046. **(A)** Projection of the number of deaths due to AA in different years for men and women of different sexes; **(B)** Prediction of the number of deaths due to AA in different age groups, genders, and years; Among them, the solid line represents the observed statistics, and the dotted line represents the prediction.

Based on the existing data, we predict the number of deaths and mortality caused by AA in the world by 2046. As can be seen from Figure A in Figure 9, the bar graph represents the number of deaths, the solid line represents the observed mortality change, and the dotted line curve represents the predicted mortality change (Supplementary Table S3). As can be seen from Figure 9, the number of deaths caused by AA increases with the increase of years, but the predicted mortality rate is decreasing year by year. By gender segmentation, AA has a greater health burden on global men than on women (Supplementary Table S4). Combined with Figure 5, it can be seen that the coefficient of APC exceeds 0 when the age is 55–59, indicating that once the age exceeds 55, the global disease burden caused by AA will increase exponentially, which can prove that AA over 55 years old is a risk factor for ASDR. Therefore, we predicted the ASDR caused by AA in men and women aged 50–94 years old. We can see the development law of ASDR. The older the age, the higher the ASDR. The number of deaths caused by AA increases year by year, but the ASDR decreases year by year, which is roughly consistent with the development law of Figure A. This may be related to the changes in global population growth, but the improvement of medical level shows that the global health burden of AA has decreased.

## 4 Discussion

This study conducted a comprehensive and systematic analysis of the changing trend of AA disease burden worldwide from 1990 to 2021, including an in-depth stratified study of populations of different genders, age groups, and regions, revealing a significant increase in the number of AA-related deaths worldwide during this period. The results of the study showed that, similar to the results of previous studies, although the number of deaths caused by AA continued to increase worldwide, thanks to advances in medical technology and treatment methods, the ASDR worldwide has generally shown a downward trend (23). This is consistent with the previous GBD study results by Wang, Z et al. (24), further verifying the effectiveness of medical progress in reducing AA mortality. However, despite the overall decline in ASDR worldwide, differences between different regions and populations remain significant, especially in areas with lower SDI. The study found that the ASDR in Low SDI and Low-middle SDI areas has been on the rise over the past 30 years. This trend reflects the inadequacy of health infrastructure, medical technology and resources in these areas, resulting in significant shortcomings in the early screening and management of AA (25, 26). In contrast, high SDI regions benefited from more developed medical systems and more complete screening and treatment methods, and their AA mortality rate dropped significantly, indicating that advanced medical resources play a key role in reducing the disease burden, Data from a 2019 GBD study showed that (27), Due to advanced medical infrastructure, improved diagnostic methods, and better access to treatment including preventive measures and surgery, AA mortality rates have significantly decreased in high SDI regions, a trend that is in stark contrast to low SDI regions. This regional inequality not only reveals the serious uneven distribution of medical resources around the world, but also highlights the weaknesses of public health systems around the world. Some studies support the view that high SDI regions, such as Oceania and high-income North America, have seen the fastest decline in AA burden. These declines are attributed to better health care access, universal screening programs, and advances in treatment, such as minimally invasive procedures like endovascular aneurysm repair (28). This suggests that early detection and timely medical intervention play a vital role in the effective management of AA, especially in areas with more advanced healthcare systems. In low-SDI areas, residents have a higher risk of death from AA due to a lack of medical resources, lack of early screening methods, and effective intervention measures. In high-SDI areas, sufficient medical resources, universal health care, advanced screening technology, and timely treatment have significantly reduced AA-related mortality.

This inequality has further exacerbated health disparities around the world. To effectively address this challenge, the international community, public health agencies, and governments must increase investment in medical infrastructure in low-SDI and low-middle SDI regions, especially in early screening, prevention, and treatment of high-risk diseases such as AA (29). Only by improving the equity of health care on a global scale can we truly reduce the GBD caused by AA and reduce the health inequality caused by resource differences (30).

Overall, this study not only highlights the continued impact of AA as an important global disease, but also calls on countries to take more proactive measures, especially in low-resource areas, to promote the popularization of medical technology and resources, narrow the health inequalities caused by socio-demographic differences, and thus reduce the global mortality rate caused by AA. This study also further shows that population aging and growth are the main factors driving the increase in deaths caused by AA worldwide, especially in areas with high SDI, where the impact of aging on the increase in deaths is particularly significant (31–33). This is closely related to the macro trend of global aging. As the global population gradually enters an aging society, the proportion of the elderly population continues to rise, and the incidence and mortality risk of aortic aneurysms also increase (34). The onset of aortic aneurysm is closely related to vascular aging. The elasticity of blood vessels in the elderly decreases and blood pressure rises, so the incidence of aortic aneurysm is higher, which in turn leads to an increase in mortality (4).

In addition, through the analysis of this study, the impact of aging is particularly evident in high SDI areas. The increase in the elderly population in these areas not only leads to an increase in the number of AA-related deaths, but also places higher demands on the public health system and medical resources. Although aging is an inevitable natural process, thanks to the advancement of medical technology in high SDI areas, the ASDR of AA has been significantly reduced. This shows that the integration of advanced imaging technologies such as magnetic resonance angiography (MRA) and computed tomography angiography (CTA) has become crucial in early detection and management. Advanced screening technology, early diagnosis, improved treatment methods, and long-term risk management can effectively reduce AA-related mortality rates (35). This discovery provides valuable experience and support for other regions in coping with the disease burden brought about by future population aging, especially in the prevention and management of high-incidence diseases such as AA, where technological progress and innovation in the medical system are crucial.

The results of the study also pointed out that men have higher ASDR than women in all age groups. This gender difference is consistent with the results of previous epidemiological studies, indicating that the incidence of AA is more common in men, resulting in a relatively higher risk of death in men. AA is closely related to a variety of risk factors, of which smoking and high blood pressure are the most critical risk factors, and these factors are more common in the male population. Smoking damages the inner wall of the blood vessels, resulting in decreased vascular elasticity and promoting the formation of AA; at the same time, high blood pressure increases the pressure on the blood vessel wall, making already damaged blood vessels more susceptible to tumorigenesis and rupture. Therefore, men have a higher exposure level to the risk of morbidity and mortality from AA (36–38).

At the same time, the study also showed that although men have higher ASDR in all age groups, women's AA-related mortality rate increases significantly with aging, especially after menopause (39). This phenomenon may be related to the decline in estrogen levels in women after menopause. There is strong evidence to support the view that estrogen plays a protective role in maintaining vascular health, particularly by enhancing vascular elasticity and regulating blood pressure. Estrogen, particularly 17-β-estradiol, exerts its cardioprotective effects through its receptors, such as estrogen receptor α (ERα) and estrogen receptor β (ERβ). These receptors help regulate vascular function and maintain cardiovascular homeostasis by promoting vasodilation, reducing arterial stiffness, and lowering blood pressure (40, 41). However, as women enter menopause, the decrease in estrogen leads to weakened vascular protection, increased blood pressure, and an increased risk of aortic aneurysm. Therefore, older women, especially postmenopausal women, should become the focus of aortic aneurysm prevention and management.

Although existing medical advances may further reduce the global ASDR, this study predicts that the number of deaths caused by AA will continue to rise in the future. This means that early screening and management of AA must be strengthened worldwide, especially in areas with limited resources and an aging population. Future policymakers should focus on these high-risk areas and promote international cooperation to ensure that all countries have access to sufficient resources to meet the challenges of aging. In addition, the predictive analysis of this study provides an important reference for public health policies, especially for the management of the global disease burden and the optimization of preventive measures.

## Data Availability

The original contributions presented in the study are included in the article/Supplementary Material, further inquiries can be directed to the corresponding author.
