# Supplementary material for: Global trends and stratified analysis of aortic aneurysm mortality: insights from the GBD 2021 study
Source: Front Cardiovasc Med. 2025 May 22;12:1496166. doi: 10.3389/fcvm.2025.1496166 (PMC12137283; doi:10.3389/fcvm.2025.1496166)
Supplement: Supplementary file 1 [file Table1.docx]

**Supplementary** **Table S1. Age-Standardized Rates (ASR) of AA Across Geographic Regions, Age Groups, and Sex Stratifications in 2021**

| location | sex | age | metric | val | upper | lower |
| --- | --- | --- | --- | --- | --- | --- |
| Global | Male | 15 to 19 | Rate | 0.04 | 0.05 | 0.03 |
| Global | Female | 15 to 19 | Rate | 0.02 | 0.03 | 0.02 |
| Global | Male | 20 to 24 | Rate | 0.08 | 0.09 | 0.07 |
| Global | Female | 20 to 24 | Rate | 0.05 | 0.06 | 0.04 |
| Global | Male | 25 to 29 | Rate | 0.14 | 0.16 | 0.12 |
| Global | Female | 25 to 29 | Rate | 0.06 | 0.07 | 0.05 |
| Global | Male | 30 to 34 | Rate | 0.25 | 0.29 | 0.22 |
| Global | Female | 30 to 34 | Rate | 0.09 | 0.10 | 0.08 |
| Global | Male | 35 to 39 | Rate | 0.45 | 0.52 | 0.39 |
| Global | Female | 35 to 39 | Rate | 0.15 | 0.16 | 0.13 |
| Global | Male | 40 to 44 | Rate | 0.76 | 0.87 | 0.68 |
| Global | Female | 40 to 44 | Rate | 0.23 | 0.26 | 0.21 |
| Global | Male | 45 to 49 | Rate | 1.18 | 1.32 | 1.07 |
| Global | Female | 45 to 49 | Rate | 0.39 | 0.44 | 0.35 |
| Global | Male | 50 to 54 | Rate | 1.81 | 2.01 | 1.66 |
| Global | Female | 50 to 54 | Rate | 0.62 | 0.70 | 0.56 |
| Global | Male | 55 to 59 | Rate | 3.08 | 3.40 | 2.82 |
| Global | Female | 55 to 59 | Rate | 1.14 | 1.29 | 1.01 |
| Global | Male | 60 to 64 | Rate | 5.37 | 5.90 | 5.01 |
| Global | Female | 60 to 64 | Rate | 2.02 | 2.30 | 1.83 |
| Global | Male | 65 to 69 | Rate | 8.67 | 9.57 | 8.04 |
| Global | Female | 65 to 69 | Rate | 3.51 | 3.86 | 3.20 |
| Global | Male | 70 to 74 | Rate | 14.32 | 15.78 | 13.43 |
| Global | Female | 70 to 74 | Rate | 6.59 | 7.36 | 5.92 |
| Global | Male | 75 to 79 | Rate | 22.24 | 24.35 | 20.60 |
| Global | Female | 75 to 79 | Rate | 11.39 | 12.44 | 10.08 |
| Global | Male | 80 to 84 | Rate | 34.72 | 37.91 | 30.99 |
| Global | Female | 80 to 84 | Rate | 19.30 | 21.35 | 16.14 |
| Global | Male | 85 to 89 | Rate | 57.94 | 62.44 | 50.41 |
| Global | Female | 85 to 89 | Rate | 34.09 | 38.92 | 26.06 |
| Global | Male | 90 to 94 | Rate | 89.29 | 97.35 | 75.27 |
| Global | Female | 90 to 94 | Rate | 57.91 | 66.94 | 41.84 |
| Global | Male | 95 plus | Rate | 100.50 | 112.24 | 76.52 |
| Global | Female | 95 plus | Rate | 89.24 | 105.90 | 60.11 |
| Low-middle SDI | Male | 15 to 19 | Rate | 0.03 | 0.04 | 0.03 |
| Low-middle SDI | Female | 15 to 19 | Rate | 0.02 | 0.03 | 0.02 |
| Low-middle SDI | Male | 20 to 24 | Rate | 0.06 | 0.08 | 0.05 |
| Low-middle SDI | Female | 20 to 24 | Rate | 0.04 | 0.06 | 0.03 |
| Low-middle SDI | Male | 25 to 29 | Rate | 0.10 | 0.13 | 0.08 |
| Low-middle SDI | Female | 25 to 29 | Rate | 0.06 | 0.08 | 0.04 |
| Low-middle SDI | Male | 30 to 34 | Rate | 0.17 | 0.25 | 0.13 |
| Low-middle SDI | Female | 30 to 34 | Rate | 0.08 | 0.11 | 0.06 |
| Low-middle SDI | Male | 35 to 39 | Rate | 0.28 | 0.40 | 0.22 |
| Low-middle SDI | Female | 35 to 39 | Rate | 0.13 | 0.17 | 0.10 |
| Low-middle SDI | Male | 40 to 44 | Rate | 0.51 | 0.75 | 0.41 |
| Low-middle SDI | Female | 40 to 44 | Rate | 0.21 | 0.27 | 0.16 |
| Low-middle SDI | Male | 45 to 49 | Rate | 0.78 | 1.14 | 0.61 |
| Low-middle SDI | Female | 45 to 49 | Rate | 0.37 | 0.48 | 0.28 |
| Low-middle SDI | Male | 50 to 54 | Rate | 1.26 | 1.85 | 0.99 |
| Low-middle SDI | Female | 50 to 54 | Rate | 0.59 | 0.78 | 0.45 |
| Low-middle SDI | Male | 55 to 59 | Rate | 2.32 | 3.50 | 1.83 |
| Low-middle SDI | Female | 55 to 59 | Rate | 1.16 | 1.51 | 0.87 |
| Low-middle SDI | Male | 60 to 64 | Rate | 3.86 | 5.76 | 3.01 |
| Low-middle SDI | Female | 60 to 64 | Rate | 1.82 | 2.40 | 1.37 |
| Low-middle SDI | Male | 65 to 69 | Rate | 6.80 | 10.37 | 5.33 |
| Low-middle SDI | Female | 65 to 69 | Rate | 3.37 | 4.42 | 2.51 |
| Low-middle SDI | Male | 70 to 74 | Rate | 11.32 | 17.23 | 8.92 |
| Low-middle SDI | Female | 70 to 74 | Rate | 6.20 | 8.07 | 4.59 |
| Low-middle SDI | Male | 75 to 79 | Rate | 16.83 | 25.32 | 13.32 |
| Low-middle SDI | Female | 75 to 79 | Rate | 9.90 | 12.68 | 7.29 |
| Low-middle SDI | Male | 80 to 84 | Rate | 23.71 | 36.44 | 18.69 |
| Low-middle SDI | Female | 80 to 84 | Rate | 13.96 | 17.91 | 10.53 |
| Low-middle SDI | Male | 85 to 89 | Rate | 29.50 | 44.80 | 22.89 |
| Low-middle SDI | Female | 85 to 89 | Rate | 20.28 | 25.69 | 15.16 |
| Low-middle SDI | Male | 90 to 94 | Rate | 36.89 | 56.74 | 27.99 |
| Low-middle SDI | Female | 90 to 94 | Rate | 25.97 | 32.74 | 19.51 |
| Low-middle SDI | Male | 95 plus | Rate | 35.41 | 52.91 | 26.35 |
| Low-middle SDI | Female | 95 plus | Rate | 25.59 | 32.19 | 17.76 |
| Low SDI | Male | 15 to 19 | Rate | 0.04 | 0.07 | 0.02 |
| Low SDI | Female | 15 to 19 | Rate | 0.03 | 0.05 | 0.02 |
| Low SDI | Male | 20 to 24 | Rate | 0.07 | 0.12 | 0.03 |
| Low SDI | Female | 20 to 24 | Rate | 0.06 | 0.10 | 0.04 |
| Low SDI | Male | 25 to 29 | Rate | 0.13 | 0.21 | 0.06 |
| Low SDI | Female | 25 to 29 | Rate | 0.09 | 0.13 | 0.05 |
| Low SDI | Male | 30 to 34 | Rate | 0.19 | 0.35 | 0.08 |
| Low SDI | Female | 30 to 34 | Rate | 0.08 | 0.13 | 0.05 |
| Low SDI | Male | 35 to 39 | Rate | 0.32 | 0.58 | 0.13 |
| Low SDI | Female | 35 to 39 | Rate | 0.13 | 0.19 | 0.08 |
| Low SDI | Male | 40 to 44 | Rate | 0.51 | 0.90 | 0.22 |
| Low SDI | Female | 40 to 44 | Rate | 0.19 | 0.31 | 0.13 |
| Low SDI | Male | 45 to 49 | Rate | 0.76 | 1.34 | 0.32 |
| Low SDI | Female | 45 to 49 | Rate | 0.39 | 0.61 | 0.26 |
| Low SDI | Male | 50 to 54 | Rate | 1.20 | 2.15 | 0.49 |
| Low SDI | Female | 50 to 54 | Rate | 0.66 | 1.05 | 0.42 |
| Low SDI | Male | 55 to 59 | Rate | 2.40 | 4.23 | 1.05 |
| Low SDI | Female | 55 to 59 | Rate | 1.35 | 2.12 | 0.87 |
| Low SDI | Male | 60 to 64 | Rate | 4.14 | 7.57 | 1.81 |
| Low SDI | Female | 60 to 64 | Rate | 2.27 | 3.69 | 1.43 |
| Low SDI | Male | 65 to 69 | Rate | 7.60 | 13.54 | 3.56 |
| Low SDI | Female | 65 to 69 | Rate | 4.13 | 6.47 | 2.62 |
| Low SDI | Male | 70 to 74 | Rate | 11.97 | 21.16 | 5.72 |
| Low SDI | Female | 70 to 74 | Rate | 7.83 | 12.02 | 5.00 |
| Low SDI | Male | 75 to 79 | Rate | 18.37 | 32.83 | 8.70 |
| Low SDI | Female | 75 to 79 | Rate | 12.50 | 19.51 | 7.92 |
| Low SDI | Male | 80 to 84 | Rate | 25.12 | 45.38 | 11.98 |
| Low SDI | Female | 80 to 84 | Rate | 17.53 | 28.90 | 10.68 |
| Low SDI | Male | 85 to 89 | Rate | 30.94 | 53.88 | 15.09 |
| Low SDI | Female | 85 to 89 | Rate | 24.68 | 39.76 | 14.94 |
| Low SDI | Male | 90 to 94 | Rate | 37.28 | 64.46 | 17.50 |
| Low SDI | Female | 90 to 94 | Rate | 30.20 | 49.62 | 17.71 |
| Low SDI | Male | 95 plus | Rate | 29.60 | 50.56 | 14.04 |
| Low SDI | Female | 95 plus | Rate | 26.23 | 43.76 | 14.67 |
| High-middle SDI | Male | 15 to 19 | Rate | 0.06 | 0.07 | 0.05 |
| High-middle SDI | Female | 15 to 19 | Rate | 0.02 | 0.03 | 0.02 |
| High-middle SDI | Male | 20 to 24 | Rate | 0.11 | 0.13 | 0.09 |
| High-middle SDI | Female | 20 to 24 | Rate | 0.04 | 0.05 | 0.03 |
| High-middle SDI | Male | 25 to 29 | Rate | 0.18 | 0.21 | 0.16 |
| High-middle SDI | Female | 25 to 29 | Rate | 0.06 | 0.07 | 0.05 |
| High SDI | Male | 15 to 19 | Rate | 0.04 | 0.04 | 0.04 |
| High SDI | Female | 15 to 19 | Rate | 0.02 | 0.02 | 0.01 |
| High SDI | Male | 20 to 24 | Rate | 0.09 | 0.09 | 0.08 |
| High SDI | Female | 20 to 24 | Rate | 0.03 | 0.04 | 0.03 |
| High SDI | Male | 25 to 29 | Rate | 0.18 | 0.19 | 0.17 |
| High SDI | Female | 25 to 29 | Rate | 0.06 | 0.06 | 0.05 |
| High SDI | Male | 30 to 34 | Rate | 0.34 | 0.36 | 0.32 |
| High SDI | Female | 30 to 34 | Rate | 0.10 | 0.10 | 0.09 |
| High SDI | Male | 35 to 39 | Rate | 0.65 | 0.68 | 0.61 |
| High SDI | Female | 35 to 39 | Rate | 0.17 | 0.17 | 0.16 |
| High SDI | Male | 40 to 44 | Rate | 1.15 | 1.21 | 1.09 |
| High SDI | Female | 40 to 44 | Rate | 0.29 | 0.31 | 0.27 |
| High SDI | Male | 45 to 49 | Rate | 1.96 | 2.05 | 1.88 |
| High SDI | Female | 45 to 49 | Rate | 0.50 | 0.52 | 0.48 |
| High SDI | Male | 50 to 54 | Rate | 2.99 | 3.11 | 2.86 |
| High SDI | Female | 50 to 54 | Rate | 0.82 | 0.88 | 0.77 |
| High SDI | Male | 55 to 59 | Rate | 4.68 | 4.87 | 4.52 |
| High SDI | Female | 55 to 59 | Rate | 1.40 | 1.51 | 1.31 |
| High SDI | Male | 60 to 64 | Rate | 7.56 | 7.86 | 7.27 |
| High SDI | Female | 60 to 64 | Rate | 2.56 | 2.78 | 2.38 |
| High SDI | Male | 65 to 69 | Rate | 12.33 | 12.85 | 11.77 |
| High SDI | Female | 65 to 69 | Rate | 4.87 | 5.22 | 4.43 |
| High SDI | Male | 70 to 74 | Rate | 20.50 | 21.30 | 19.40 |
| High SDI | Female | 70 to 74 | Rate | 9.96 | 11.13 | 8.86 |
| High SDI | Male | 75 to 79 | Rate | 34.11 | 35.67 | 31.87 |
| High SDI | Female | 75 to 79 | Rate | 18.83 | 20.30 | 16.07 |
| High SDI | Male | 80 to 84 | Rate | 54.13 | 57.54 | 46.83 |
| High SDI | Female | 80 to 84 | Rate | 32.33 | 37.42 | 24.87 |
| High SDI | Male | 85 to 89 | Rate | 94.96 | 102.12 | 81.36 |
| High SDI | Female | 85 to 89 | Rate | 58.83 | 68.84 | 42.45 |
| High SDI | Male | 90 to 94 | Rate | 143.67 | 155.53 | 119.86 |
| High SDI | Female | 90 to 94 | Rate | 97.15 | 113.98 | 67.99 |
| High SDI | Male | 95 plus | Rate | 152.49 | 171.00 | 115.69 |
| High SDI | Female | 95 plus | Rate | 140.04 | 167.55 | 92.82 |
| Middle SDI | Male | 15 to 19 | Rate | 0.04 | 0.05 | 0.04 |
| Middle SDI | Female | 15 to 19 | Rate | 0.02 | 0.03 | 0.02 |
| Middle SDI | Male | 20 to 24 | Rate | 0.09 | 0.10 | 0.07 |
| Middle SDI | Female | 20 to 24 | Rate | 0.04 | 0.05 | 0.04 |
| Middle SDI | Male | 25 to 29 | Rate | 0.13 | 0.15 | 0.12 |
| Middle SDI | Female | 25 to 29 | Rate | 0.06 | 0.07 | 0.05 |
| Middle SDI | Male | 30 to 34 | Rate | 0.23 | 0.26 | 0.20 |
| Middle SDI | Female | 30 to 34 | Rate | 0.08 | 0.10 | 0.07 |
| Middle SDI | Male | 35 to 39 | Rate | 0.39 | 0.46 | 0.33 |
| Middle SDI | Female | 35 to 39 | Rate | 0.14 | 0.17 | 0.13 |
| Middle SDI | Male | 40 to 44 | Rate | 0.61 | 0.71 | 0.52 |
| Middle SDI | Female | 40 to 44 | Rate | 0.22 | 0.25 | 0.19 |
| Middle SDI | Male | 45 to 49 | Rate | 0.86 | 1.00 | 0.74 |
| Middle SDI | Female | 45 to 49 | Rate | 0.34 | 0.40 | 0.30 |
| Middle SDI | Male | 50 to 54 | Rate | 1.30 | 1.49 | 1.13 |
| Middle SDI | Female | 50 to 54 | Rate | 0.50 | 0.56 | 0.45 |
| Middle SDI | Male | 55 to 59 | Rate | 2.13 | 2.49 | 1.84 |
| Middle SDI | Female | 55 to 59 | Rate | 0.90 | 1.04 | 0.77 |
| Middle SDI | Male | 60 to 64 | Rate | 3.70 | 4.15 | 3.30 |
| Middle SDI | Female | 60 to 64 | Rate | 1.59 | 1.77 | 1.43 |
| Middle SDI | Male | 65 to 69 | Rate | 5.65 | 6.43 | 5.01 |
| Middle SDI | Female | 65 to 69 | Rate | 2.54 | 2.84 | 2.27 |
| Middle SDI | Male | 70 to 74 | Rate | 9.28 | 10.48 | 8.27 |
| Middle SDI | Female | 70 to 74 | Rate | 4.30 | 4.83 | 3.81 |
| Middle SDI | Male | 75 to 79 | Rate | 14.31 | 16.08 | 12.68 |
| Middle SDI | Female | 75 to 79 | Rate | 7.18 | 8.07 | 6.32 |
| Middle SDI | Male | 80 to 84 | Rate | 20.49 | 23.09 | 18.15 |
| Middle SDI | Female | 80 to 84 | Rate | 11.19 | 12.58 | 9.44 |
| Middle SDI | Male | 85 to 89 | Rate | 31.18 | 35.64 | 26.66 |
| Middle SDI | Female | 85 to 89 | Rate | 16.98 | 19.47 | 13.97 |
| Middle SDI | Male | 90 to 94 | Rate | 42.60 | 49.23 | 35.03 |
| Middle SDI | Female | 90 to 94 | Rate | 23.84 | 28.14 | 18.49 |
| Middle SDI | Male | 95 plus | Rate | 46.61 | 56.28 | 34.39 |
| Middle SDI | Female | 95 plus | Rate | 33.23 | 41.70 | 23.75 |
| High-middle SDI | Male | 30 to 34 | Rate | 0.36 | 0.41 | 0.31 |
| High-middle SDI | Female | 30 to 34 | Rate | 0.09 | 0.10 | 0.08 |
| High-middle SDI | Male | 35 to 39 | Rate | 0.67 | 0.78 | 0.57 |
| High-middle SDI | Female | 35 to 39 | Rate | 0.16 | 0.19 | 0.14 |
| High-middle SDI | Male | 40 to 44 | Rate | 1.16 | 1.34 | 1.00 |
| High-middle SDI | Female | 40 to 44 | Rate | 0.27 | 0.31 | 0.23 |
| High-middle SDI | Male | 45 to 49 | Rate | 1.69 | 1.90 | 1.51 |
| High-middle SDI | Female | 45 to 49 | Rate | 0.42 | 0.48 | 0.37 |
| High-middle SDI | Male | 50 to 54 | Rate | 2.41 | 2.69 | 2.14 |
| High-middle SDI | Female | 50 to 54 | Rate | 0.68 | 0.80 | 0.59 |
| High-middle SDI | Male | 55 to 59 | Rate | 4.01 | 4.46 | 3.58 |
| High-middle SDI | Female | 55 to 59 | Rate | 1.22 | 1.44 | 1.03 |
| High-middle SDI | Male | 60 to 64 | Rate | 7.17 | 7.75 | 6.60 |
| High-middle SDI | Female | 60 to 64 | Rate | 2.20 | 2.65 | 1.92 |
| High-middle SDI | Male | 65 to 69 | Rate | 10.93 | 11.86 | 10.06 |
| High-middle SDI | Female | 65 to 69 | Rate | 3.51 | 3.94 | 3.15 |
| High-middle SDI | Male | 70 to 74 | Rate | 16.55 | 17.80 | 15.30 |
| High-middle SDI | Female | 70 to 74 | Rate | 5.78 | 6.72 | 5.11 |
| High-middle SDI | Male | 75 to 79 | Rate | 23.07 | 25.24 | 21.08 |
| High-middle SDI | Female | 75 to 79 | Rate | 8.62 | 9.46 | 7.61 |
| High-middle SDI | Male | 80 to 84 | Rate | 34.27 | 36.91 | 31.37 |
| High-middle SDI | Female | 80 to 84 | Rate | 15.12 | 16.44 | 13.02 |
| High-middle SDI | Male | 85 to 89 | Rate | 49.22 | 53.73 | 43.59 |
| High-middle SDI | Female | 85 to 89 | Rate | 22.26 | 24.80 | 18.48 |
| High-middle SDI | Male | 90 to 94 | Rate | 68.15 | 74.32 | 57.83 |
| High-middle SDI | Female | 90 to 94 | Rate | 31.00 | 34.59 | 24.35 |
| High-middle SDI | Male | 95 plus | Rate | 77.66 | 87.15 | 61.13 |
| High-middle SDI | Female | 95 plus | Rate | 36.71 | 41.63 | 27.42 |

**Supplementary** **Table S2. Age-period-cohort model and mortality coefficient for Deaths**

| age | coef | lower | upper | apc_name |
| --- | --- | --- | --- | --- |
| 15 to 19 | -3.00 | -3.05 | -2.94 | Age |
| 20 to 24 | -2.52 | -2.56 | -2.48 | Age |
| 25 to 29 | -2.19 | -2.22 | -2.16 | Age |
| 30 to 34 | -1.82 | -1.85 | -1.79 | Age |
| 35 to 39 | -1.39 | -1.41 | -1.36 | Age |
| 40 to 44 | -1.00 | -1.02 | -0.97 | Age |
| 45 to 49 | -0.60 | -0.62 | -0.58 | Age |
| 50 to 54 | -0.25 | -0.27 | -0.24 | Age |
| 55 to 59 | 0.14 | 0.13 | 0.16 | Age |
| 60 to 64 | 0.51 | 0.50 | 0.52 | Age |
| 65 to 69 | 0.89 | 0.89 | 0.90 | Age |
| 70 to 74 | 1.22 | 1.22 | 1.23 | Age |
| 75 to 79 | 1.54 | 1.53 | 1.55 | Age |
| 80 to 84 | 1.78 | 1.78 | 1.79 | Age |
| 85 to 89 | 2.07 | 2.06 | 2.08 | Age |
| 90 to 94 | 2.27 | 2.26 | 2.29 | Age |
| 95 to 99 | 2.34 | 2.32 | 2.36 | Age |
| 1897 to 1901 | 1.57 | 1.51 | 1.62 | Cohort |
| 1902 to 1906 | 1.46 | 1.43 | 1.49 | Cohort |
| 1907 to 1911 | 1.37 | 1.35 | 1.39 | Cohort |
| 1912 to 1916 | 1.30 | 1.29 | 1.32 | Cohort |
| 1917 to 1921 | 1.15 | 1.14 | 1.17 | Cohort |
| 1922 to 1926 | 1.00 | 0.98 | 1.01 | Cohort |
| 1927 to 1931 | 0.79 | 0.78 | 0.80 | Cohort |
| 1932 to 1936 | 0.56 | 0.54 | 0.57 | Cohort |
| 1937 to 1941 | 0.33 | 0.31 | 0.34 | Cohort |
| 1942 to 1946 | 0.13 | 0.12 | 0.15 | Cohort |
| 1947 to 1951 | -0.05 | -0.06 | -0.03 | Cohort |
| 1952 to 1956 | -0.20 | -0.22 | -0.19 | Cohort |
| 1957 to 1961 | -0.34 | -0.36 | -0.32 | Cohort |
| 1962 to 1966 | -0.50 | -0.52 | -0.47 | Cohort |
| 1967 to 1971 | -0.66 | -0.69 | -0.64 | Cohort |
| 1972 to 1976 | -0.77 | -0.79 | -0.74 | Cohort |
| 1977 to 1981 | -0.85 | -0.88 | -0.82 | Cohort |
| 1982 to 1986 | -0.97 | -1.00 | -0.93 | Cohort |
| 1987 to 1991 | -1.11 | -1.15 | -1.07 | Cohort |
| 1992 to 1996 | -1.25 | -1.31 | -1.20 | Cohort |
| 1997 to 2001 | -1.40 | -1.47 | -1.32 | Cohort |
| 2002 to 2006 | -1.56 | -1.70 | -1.42 | Cohort |
| 1992 to 1996 | -0.28 | -0.29 | -0.27 | Period |
| 1997 to 2001 | -0.15 | -0.16 | -0.15 | Period |
| 2002 to 2006 | -0.06 | -0.06 | -0.05 | Period |
| 2007 to 2011 | 0.03 | 0.03 | 0.04 | Period |
| 2012 to 2016 | 0.15 | 0.15 | 0.16 | Period |
| 2017 to 2021 | 0.30 | 0.30 | 0.31 | Period |

**Supplementary** **Table S3. Global Projection of ASR and Deaths Attributable to AA Until 2046**

| sex | year | ASR | case |
| --- | --- | --- | --- |
| Male | 1992 | 5.47 | 61319 |
| Male | 1993 | 5.54 | 63908 |
| Male | 1994 | 5.52 | 65529 |
| Male | 1995 | 5.52 | 67188 |
| Male | 1996 | 5.45 | 67904 |
| Male | 1997 | 5.35 | 68196 |
| Male | 1998 | 5.27 | 68923 |
| Male | 1999 | 5.25 | 70523 |
| Male | 2000 | 5.14 | 70982 |
| Male | 2001 | 5.00 | 70769 |
| Male | 2002 | 4.93 | 71802 |
| Male | 2003 | 4.86 | 72666 |
| Male | 2004 | 4.73 | 72799 |
| Male | 2005 | 4.65 | 73763 |
| Male | 2006 | 4.52 | 73871 |
| Male | 2007 | 4.43 | 74751 |
| Male | 2008 | 4.33 | 75479 |
| Male | 2009 | 4.21 | 75794 |
| Male | 2010 | 4.14 | 76930 |
| Male | 2011 | 4.03 | 77204 |
| Male | 2012 | 3.93 | 77926 |
| Male | 2013 | 3.86 | 78949 |
| Male | 2014 | 3.82 | 80646 |
| Male | 2015 | 3.79 | 82576 |
| Male | 2016 | 3.78 | 85054 |
| Male | 2017 | 3.74 | 86727 |
| Male | 2018 | 3.71 | 88951 |
| Male | 2019 | 3.67 | 90946 |
| Male | 2020 | 3.62 | 92175 |
| Male | 2021 | 3.60 | 93864 |
| Male | 2022 | 3.57 | 97961 |
| Male | 2023 | 3.54 | 100130 |
| Male | 2024 | 3.51 | 102502 |
| Male | 2025 | 3.50 | 105528 |
| Male | 2026 | 3.48 | 108546 |
| Male | 2027 | 3.47 | 111482 |
| Male | 2028 | 3.46 | 114491 |
| Male | 2029 | 3.45 | 117685 |
| Male | 2030 | 3.46 | 121461 |
| Male | 2031 | 3.46 | 125257 |
| Male | 2032 | 3.47 | 128999 |
| Male | 2033 | 3.47 | 132808 |
| Male | 2034 | 3.48 | 136783 |
| Male | 2035 | 3.49 | 141317 |
| Male | 2036 | 3.51 | 145868 |
| Male | 2037 | 3.52 | 150365 |
| Male | 2038 | 3.54 | 154927 |
| Male | 2039 | 3.56 | 159649 |
| Male | 2040 | 3.58 | 164686 |
| Male | 2041 | 3.60 | 169721 |
| Male | 2042 | 3.62 | 174679 |
| Male | 2043 | 3.64 | 179661 |
| Male | 2044 | 3.66 | 184764 |
| Male | 2045 | 3.68 | 189917 |
| Male | 2046 | 3.70 | 195056 |
| Female | 1992 | 2.25 | 32929 |
| Female | 1993 | 2.27 | 34150 |
| Female | 1994 | 2.30 | 35522 |
| Female | 1995 | 2.31 | 36453 |
| Female | 1996 | 2.29 | 37152 |
| Female | 1997 | 2.26 | 37568 |
| Female | 1998 | 2.26 | 38415 |
| Female | 1999 | 2.31 | 40348 |
| Female | 2000 | 2.28 | 40909 |
| Female | 2001 | 2.20 | 40427 |
| Female | 2002 | 2.16 | 40829 |
| Female | 2003 | 2.14 | 41502 |
| Female | 2004 | 2.11 | 42119 |
| Female | 2005 | 2.08 | 42724 |
| Female | 2006 | 2.04 | 43146 |
| Female | 2007 | 2.02 | 44000 |
| Female | 2008 | 1.98 | 44439 |
| Female | 2009 | 1.95 | 45162 |
| Female | 2010 | 1.93 | 46036 |
| Female | 2011 | 1.91 | 47002 |
| Female | 2012 | 1.89 | 47972 |
| Female | 2013 | 1.87 | 49111 |
| Female | 2014 | 1.87 | 50588 |
| Female | 2015 | 1.87 | 52147 |
| Female | 2016 | 1.88 | 54054 |
| Female | 2017 | 1.86 | 55256 |
| Female | 2018 | 1.84 | 56562 |
| Female | 2019 | 1.84 | 58066 |
| Female | 2020 | 1.80 | 58673 |
| Female | 2021 | 1.80 | 60063 |
| Female | 2022 | 1.80 | 62451 |
| Female | 2023 | 1.79 | 63981 |
| Female | 2024 | 1.78 | 65718 |
| Female | 2025 | 1.78 | 67676 |
| Female | 2026 | 1.77 | 69642 |
| Female | 2027 | 1.77 | 71557 |
| Female | 2028 | 1.76 | 73560 |
| Female | 2029 | 1.76 | 75745 |
| Female | 2030 | 1.76 | 78128 |
| Female | 2031 | 1.75 | 80551 |
| Female | 2032 | 1.75 | 82960 |
| Female | 2033 | 1.75 | 85458 |
| Female | 2034 | 1.74 | 88125 |
| Female | 2035 | 1.74 | 90959 |
| Female | 2036 | 1.74 | 93818 |
| Female | 2037 | 1.74 | 96638 |
| Female | 2038 | 1.74 | 99523 |
| Female | 2039 | 1.73 | 102553 |
| Female | 2040 | 1.73 | 105697 |
| Female | 2041 | 1.73 | 108818 |
| Female | 2042 | 1.73 | 111843 |
| Female | 2043 | 1.73 | 114871 |
| Female | 2044 | 1.73 | 117987 |
| Female | 2045 | 1.73 | 121120 |
| Female | 2046 | 1.73 | 124206 |
| Both | 1992 | 3.60 | 94248 |
| Both | 1993 | 3.65 | 98058 |
| Both | 1994 | 3.67 | 101051 |
| Both | 1995 | 3.67 | 103641 |
| Both | 1996 | 3.64 | 105057 |
| Both | 1997 | 3.57 | 105764 |
| Both | 1998 | 3.54 | 107338 |
| Both | 1999 | 3.57 | 110872 |
| Both | 2000 | 3.51 | 111892 |
| Both | 2001 | 3.40 | 111197 |
| Both | 2002 | 3.35 | 112631 |
| Both | 2003 | 3.31 | 114168 |
| Both | 2004 | 3.24 | 114918 |
| Both | 2005 | 3.19 | 116487 |
| Both | 2006 | 3.12 | 117017 |
| Both | 2007 | 3.07 | 118750 |
| Both | 2008 | 3.00 | 119918 |
| Both | 2009 | 2.94 | 120956 |
| Both | 2010 | 2.90 | 122966 |
| Both | 2011 | 2.84 | 124205 |
| Both | 2012 | 2.79 | 125898 |
| Both | 2013 | 2.75 | 128059 |
| Both | 2014 | 2.73 | 131234 |
| Both | 2015 | 2.73 | 134723 |
| Both | 2016 | 2.73 | 139108 |
| Both | 2017 | 2.70 | 141983 |
| Both | 2018 | 2.68 | 145513 |
| Both | 2019 | 2.66 | 149012 |
| Both | 2020 | 2.62 | 150848 |
| Both | 2021 | 2.61 | 153927 |
| Both | 2022 | 2.60 | 160412 |
| Both | 2023 | 2.59 | 164111 |
| Both | 2024 | 2.57 | 168220 |
| Both | 2025 | 2.56 | 173204 |
| Both | 2026 | 2.56 | 178188 |
| Both | 2027 | 2.55 | 183039 |
| Both | 2028 | 2.55 | 188051 |
| Both | 2029 | 2.54 | 193430 |
| Both | 2030 | 2.54 | 199589 |
| Both | 2031 | 2.54 | 205808 |
| Both | 2032 | 2.54 | 211958 |
| Both | 2033 | 2.54 | 218265 |
| Both | 2034 | 2.55 | 224908 |
| Both | 2035 | 2.55 | 232276 |
| Both | 2036 | 2.56 | 239686 |
| Both | 2037 | 2.57 | 247003 |
| Both | 2038 | 2.58 | 254450 |
| Both | 2039 | 2.58 | 262202 |
| Both | 2040 | 2.59 | 270383 |
| Both | 2041 | 2.60 | 278538 |
| Both | 2042 | 2.61 | 286522 |
| Both | 2043 | 2.62 | 294532 |
| Both | 2044 | 2.63 | 302751 |
| Both | 2045 | 2.64 | 311036 |
| Both | 2046 | 2.65 | 319262 |

**Supplementary** **Table S4. Global Projections of Future Mortality Rates and Deaths Attributable to AA Across Age Groups Until 2046**

| sex | age | year | rate | case |
| --- | --- | --- | --- | --- |
| Male | 50 to 54 | 1992 | 2.14 | 2339 |
| Male | 50 to 54 | 1993 | 2.22 | 2442 |
| Male | 50 to 54 | 1994 | 2.23 | 2459 |
| Male | 50 to 54 | 1995 | 2.20 | 2440 |
| Male | 50 to 54 | 1996 | 2.14 | 2398 |
| Male | 50 to 54 | 1997 | 2.08 | 2422 |
| Male | 50 to 54 | 1998 | 2.06 | 2492 |
| Male | 50 to 54 | 1999 | 2.09 | 2652 |
| Male | 50 to 54 | 2000 | 2.16 | 2851 |
| Male | 50 to 54 | 2001 | 2.17 | 3001 |
| Male | 50 to 54 | 2002 | 2.18 | 3108 |
| Male | 50 to 54 | 2003 | 2.16 | 3196 |
| Male | 50 to 54 | 2004 | 2.11 | 3210 |
| Male | 50 to 54 | 2005 | 2.12 | 3341 |
| Male | 50 to 54 | 2006 | 2.07 | 3349 |
| Male | 50 to 54 | 2007 | 2.05 | 3391 |
| Male | 50 to 54 | 2008 | 2.06 | 3453 |
| Male | 50 to 54 | 2009 | 2.03 | 3433 |
| Male | 50 to 54 | 2010 | 2.02 | 3451 |
| Male | 50 to 54 | 2011 | 2.00 | 3477 |
| Male | 50 to 54 | 2012 | 1.97 | 3506 |
| Male | 50 to 54 | 2013 | 1.93 | 3558 |
| Male | 50 to 54 | 2014 | 1.92 | 3669 |
| Male | 50 to 54 | 2015 | 1.89 | 3745 |
| Male | 50 to 54 | 2016 | 1.89 | 3865 |
| Male | 50 to 54 | 2017 | 1.85 | 3868 |
| Male | 50 to 54 | 2018 | 1.84 | 3927 |
| Male | 50 to 54 | 2019 | 1.85 | 3997 |
| Male | 50 to 54 | 2020 | 1.81 | 3970 |
| Male | 50 to 54 | 2021 | 1.81 | 4021 |
| Male | 50 to 54 | 2022 | 1.91 | 4376 |
| Male | 50 to 54 | 2023 | 1.93 | 4473 |
| Male | 50 to 54 | 2024 | 1.96 | 4557 |
| Male | 50 to 54 | 2025 | 1.99 | 4645 |
| Male | 50 to 54 | 2026 | 2.02 | 4735 |
| Male | 50 to 54 | 2027 | 2.05 | 4835 |
| Male | 50 to 54 | 2028 | 2.07 | 4942 |
| Male | 50 to 54 | 2029 | 2.10 | 5050 |
| Male | 50 to 54 | 2030 | 2.13 | 5154 |
| Male | 50 to 54 | 2031 | 2.15 | 5270 |
| Male | 50 to 54 | 2032 | 2.18 | 5404 |
| Male | 50 to 54 | 2033 | 2.20 | 5567 |
| Male | 50 to 54 | 2034 | 2.23 | 5756 |
| Male | 50 to 54 | 2035 | 2.25 | 5953 |
| Male | 50 to 54 | 2036 | 2.28 | 6146 |
| Male | 50 to 54 | 2037 | 2.30 | 6318 |
| Male | 50 to 54 | 2038 | 2.32 | 6469 |
| Male | 50 to 54 | 2039 | 2.35 | 6609 |
| Male | 50 to 54 | 2040 | 2.37 | 6728 |
| Male | 50 to 54 | 2041 | 2.39 | 6831 |
| Male | 50 to 54 | 2042 | 2.41 | 6914 |
| Male | 50 to 54 | 2043 | 2.43 | 6969 |
| Male | 50 to 54 | 2044 | 2.46 | 6999 |
| Male | 50 to 54 | 2045 | 2.48 | 7021 |
| Male | 50 to 54 | 2046 | 2.50 | 7052 |
| Male | 55 to 59 | 1992 | 3.78 | 3612 |
| Male | 55 to 59 | 1993 | 3.96 | 3843 |
| Male | 55 to 59 | 1994 | 4.13 | 4060 |
| Male | 55 to 59 | 1995 | 4.20 | 4181 |
| Male | 55 to 59 | 1996 | 4.13 | 4147 |
| Male | 55 to 59 | 1997 | 4.01 | 4044 |
| Male | 55 to 59 | 1998 | 3.89 | 3947 |
| Male | 55 to 59 | 1999 | 3.86 | 3936 |
| Male | 55 to 59 | 2000 | 3.81 | 3900 |
| Male | 55 to 59 | 2001 | 3.71 | 3859 |
| Male | 55 to 59 | 2002 | 3.69 | 3996 |
| Male | 55 to 59 | 2003 | 3.67 | 4128 |
| Male | 55 to 59 | 2004 | 3.61 | 4288 |
| Male | 55 to 59 | 2005 | 3.72 | 4609 |
| Male | 55 to 59 | 2006 | 3.69 | 4803 |
| Male | 55 to 59 | 2007 | 3.64 | 4899 |
| Male | 55 to 59 | 2008 | 3.58 | 4997 |
| Male | 55 to 59 | 2009 | 3.46 | 4967 |
| Male | 55 to 59 | 2010 | 3.38 | 5051 |
| Male | 55 to 59 | 2011 | 3.33 | 5110 |
| Male | 55 to 59 | 2012 | 3.29 | 5162 |
| Male | 55 to 59 | 2013 | 3.28 | 5212 |
| Male | 55 to 59 | 2014 | 3.31 | 5332 |
| Male | 55 to 59 | 2015 | 3.34 | 5426 |
| Male | 55 to 59 | 2016 | 3.38 | 5592 |
| Male | 55 to 59 | 2017 | 3.32 | 5631 |
| Male | 55 to 59 | 2018 | 3.32 | 5818 |
| Male | 55 to 59 | 2019 | 3.24 | 5909 |
| Male | 55 to 59 | 2020 | 3.13 | 5926 |
| Male | 55 to 59 | 2021 | 3.08 | 6002 |
| Male | 55 to 59 | 2022 | 3.21 | 6483 |
| Male | 55 to 59 | 2023 | 3.21 | 6636 |
| Male | 55 to 59 | 2024 | 3.21 | 6766 |
| Male | 55 to 59 | 2025 | 3.24 | 6948 |
| Male | 55 to 59 | 2026 | 3.28 | 7116 |
| Male | 55 to 59 | 2027 | 3.31 | 7274 |
| Male | 55 to 59 | 2028 | 3.34 | 7411 |
| Male | 55 to 59 | 2029 | 3.37 | 7526 |
| Male | 55 to 59 | 2030 | 3.42 | 7675 |
| Male | 55 to 59 | 2031 | 3.48 | 7827 |
| Male | 55 to 59 | 2032 | 3.53 | 7996 |
| Male | 55 to 59 | 2033 | 3.58 | 8177 |
| Male | 55 to 59 | 2034 | 3.63 | 8360 |
| Male | 55 to 59 | 2035 | 3.68 | 8539 |
| Male | 55 to 59 | 2036 | 3.72 | 8738 |
| Male | 55 to 59 | 2037 | 3.77 | 8967 |
| Male | 55 to 59 | 2038 | 3.81 | 9245 |
| Male | 55 to 59 | 2039 | 3.86 | 9568 |
| Male | 55 to 59 | 2040 | 3.89 | 9889 |
| Male | 55 to 59 | 2041 | 3.93 | 10202 |
| Male | 55 to 59 | 2042 | 3.97 | 10480 |
| Male | 55 to 59 | 2043 | 4.00 | 10723 |
| Male | 55 to 59 | 2044 | 4.04 | 10948 |
| Male | 55 to 59 | 2045 | 4.08 | 11149 |
| Male | 55 to 59 | 2046 | 4.11 | 11322 |
| Male | 60 to 64 | 1992 | 7.49 | 6125 |
| Male | 60 to 64 | 1993 | 7.59 | 6287 |
| Male | 60 to 64 | 1994 | 7.57 | 6330 |
| Male | 60 to 64 | 1995 | 7.47 | 6307 |
| Male | 60 to 64 | 1996 | 7.23 | 6189 |
| Male | 60 to 64 | 1997 | 7.01 | 6099 |
| Male | 60 to 64 | 1998 | 6.91 | 6111 |
| Male | 60 to 64 | 1999 | 6.98 | 6261 |
| Male | 60 to 64 | 2000 | 6.95 | 6325 |
| Male | 60 to 64 | 2001 | 6.79 | 6246 |
| Male | 60 to 64 | 2002 | 6.72 | 6234 |
| Male | 60 to 64 | 2003 | 6.62 | 6188 |
| Male | 60 to 64 | 2004 | 6.39 | 6033 |
| Male | 60 to 64 | 2005 | 6.26 | 5973 |
| Male | 60 to 64 | 2006 | 6.02 | 5860 |
| Male | 60 to 64 | 2007 | 5.96 | 6058 |
| Male | 60 to 64 | 2008 | 5.90 | 6250 |
| Male | 60 to 64 | 2009 | 5.80 | 6502 |
| Male | 60 to 64 | 2010 | 5.85 | 6847 |
| Male | 60 to 64 | 2011 | 5.80 | 7129 |
| Male | 60 to 64 | 2012 | 5.64 | 7180 |
| Male | 60 to 64 | 2013 | 5.52 | 7275 |
| Male | 60 to 64 | 2014 | 5.44 | 7389 |
| Male | 60 to 64 | 2015 | 5.37 | 7589 |
| Male | 60 to 64 | 2016 | 5.40 | 7841 |
| Male | 60 to 64 | 2017 | 5.35 | 7934 |
| Male | 60 to 64 | 2018 | 5.44 | 8189 |
| Male | 60 to 64 | 2019 | 5.47 | 8317 |
| Male | 60 to 64 | 2020 | 5.40 | 8295 |
| Male | 60 to 64 | 2021 | 5.37 | 8346 |
| Male | 60 to 64 | 2022 | 5.38 | 8583 |
| Male | 60 to 64 | 2023 | 5.37 | 8821 |
| Male | 60 to 64 | 2024 | 5.36 | 9129 |
| Male | 60 to 64 | 2025 | 5.36 | 9484 |
| Male | 60 to 64 | 2026 | 5.36 | 9830 |
| Male | 60 to 64 | 2027 | 5.36 | 10129 |
| Male | 60 to 64 | 2028 | 5.36 | 10373 |
| Male | 60 to 64 | 2029 | 5.35 | 10583 |
| Male | 60 to 64 | 2030 | 5.41 | 10880 |
| Male | 60 to 64 | 2031 | 5.47 | 11155 |
| Male | 60 to 64 | 2032 | 5.53 | 11412 |
| Male | 60 to 64 | 2033 | 5.58 | 11638 |
| Male | 60 to 64 | 2034 | 5.64 | 11829 |
| Male | 60 to 64 | 2035 | 5.73 | 12067 |
| Male | 60 to 64 | 2036 | 5.82 | 12312 |
| Male | 60 to 64 | 2037 | 5.90 | 12584 |
| Male | 60 to 64 | 2038 | 5.99 | 12873 |
| Male | 60 to 64 | 2039 | 6.08 | 13167 |
| Male | 60 to 64 | 2040 | 6.14 | 13437 |
| Male | 60 to 64 | 2041 | 6.21 | 13740 |
| Male | 60 to 64 | 2042 | 6.28 | 14091 |
| Male | 60 to 64 | 2043 | 6.35 | 14520 |
| Male | 60 to 64 | 2044 | 6.41 | 15018 |
| Male | 60 to 64 | 2045 | 6.48 | 15546 |
| Male | 60 to 64 | 2046 | 6.55 | 16061 |
| Male | 65 to 69 | 1992 | 14.50 | 8906 |
| Male | 65 to 69 | 1993 | 14.70 | 9354 |
| Male | 65 to 69 | 1994 | 14.68 | 9644 |
| Male | 65 to 69 | 1995 | 14.59 | 9855 |
| Male | 65 to 69 | 1996 | 14.16 | 9790 |
| Male | 65 to 69 | 1997 | 13.73 | 9645 |
| Male | 65 to 69 | 1998 | 13.32 | 9480 |
| Male | 65 to 69 | 1999 | 13.08 | 9420 |
| Male | 65 to 69 | 2000 | 12.62 | 9208 |
| Male | 65 to 69 | 2001 | 12.04 | 8927 |
| Male | 65 to 69 | 2002 | 11.84 | 8952 |
| Male | 65 to 69 | 2003 | 11.59 | 8941 |
| Male | 65 to 69 | 2004 | 11.30 | 8877 |
| Male | 65 to 69 | 2005 | 11.17 | 8921 |
| Male | 65 to 69 | 2006 | 10.87 | 8820 |
| Male | 65 to 69 | 2007 | 10.69 | 8790 |
| Male | 65 to 69 | 2008 | 10.39 | 8665 |
| Male | 65 to 69 | 2009 | 10.13 | 8569 |
| Male | 65 to 69 | 2010 | 9.88 | 8474 |
| Male | 65 to 69 | 2011 | 9.51 | 8345 |
| Male | 65 to 69 | 2012 | 9.21 | 8456 |
| Male | 65 to 69 | 2013 | 9.08 | 8723 |
| Male | 65 to 69 | 2014 | 9.01 | 9187 |
| Male | 65 to 69 | 2015 | 9.17 | 9766 |
| Male | 65 to 69 | 2016 | 9.32 | 10442 |
| Male | 65 to 69 | 2017 | 9.27 | 10759 |
| Male | 65 to 69 | 2018 | 9.22 | 11103 |
| Male | 65 to 69 | 2019 | 9.11 | 11290 |
| Male | 65 to 69 | 2020 | 8.84 | 11391 |
| Male | 65 to 69 | 2021 | 8.67 | 11426 |
| Male | 65 to 69 | 2022 | 8.97 | 12027 |
| Male | 65 to 69 | 2023 | 8.95 | 12222 |
| Male | 65 to 69 | 2024 | 8.94 | 12346 |
| Male | 65 to 69 | 2025 | 8.93 | 12466 |
| Male | 65 to 69 | 2026 | 8.93 | 12626 |
| Male | 65 to 69 | 2027 | 8.92 | 12877 |
| Male | 65 to 69 | 2028 | 8.91 | 13263 |
| Male | 65 to 69 | 2029 | 8.91 | 13758 |
| Male | 65 to 69 | 2030 | 8.92 | 14329 |
| Male | 65 to 69 | 2031 | 8.93 | 14887 |
| Male | 65 to 69 | 2032 | 8.94 | 15372 |
| Male | 65 to 69 | 2033 | 8.95 | 15775 |
| Male | 65 to 69 | 2034 | 8.97 | 16129 |
| Male | 65 to 69 | 2035 | 9.07 | 16600 |
| Male | 65 to 69 | 2036 | 9.17 | 17038 |
| Male | 65 to 69 | 2037 | 9.27 | 17448 |
| Male | 65 to 69 | 2038 | 9.37 | 17809 |
| Male | 65 to 69 | 2039 | 9.47 | 18119 |
| Male | 65 to 69 | 2040 | 9.60 | 18467 |
| Male | 65 to 69 | 2041 | 9.73 | 18826 |
| Male | 65 to 69 | 2042 | 9.86 | 19226 |
| Male | 65 to 69 | 2043 | 9.99 | 19653 |
| Male | 65 to 69 | 2044 | 10.12 | 20086 |
| Male | 65 to 69 | 2045 | 10.25 | 20549 |
| Male | 65 to 69 | 2046 | 10.38 | 21065 |
| Male | 70 to 74 | 1992 | 25.09 | 10201 |
| Male | 70 to 74 | 1993 | 25.71 | 10893 |
| Male | 70 to 74 | 1994 | 25.94 | 11441 |
| Male | 70 to 74 | 1995 | 25.72 | 11772 |
| Male | 70 to 74 | 1996 | 25.01 | 11857 |
| Male | 70 to 74 | 1997 | 24.13 | 11866 |
| Male | 70 to 74 | 1998 | 23.50 | 12007 |
| Male | 70 to 74 | 1999 | 23.19 | 12276 |
| Male | 70 to 74 | 2000 | 22.33 | 12195 |
| Male | 70 to 74 | 2001 | 21.26 | 11929 |
| Male | 70 to 74 | 2002 | 20.77 | 11888 |
| Male | 70 to 74 | 2003 | 20.09 | 11690 |
| Male | 70 to 74 | 2004 | 19.25 | 11380 |
| Male | 70 to 74 | 2005 | 18.63 | 11210 |
| Male | 70 to 74 | 2006 | 17.75 | 10914 |
| Male | 70 to 74 | 2007 | 17.33 | 10920 |
| Male | 70 to 74 | 2008 | 16.96 | 10947 |
| Male | 70 to 74 | 2009 | 16.55 | 10922 |
| Male | 70 to 74 | 2010 | 16.31 | 10986 |
| Male | 70 to 74 | 2011 | 15.79 | 10843 |
| Male | 70 to 74 | 2012 | 15.42 | 10758 |
| Male | 70 to 74 | 2013 | 14.96 | 10627 |
| Male | 70 to 74 | 2014 | 14.69 | 10632 |
| Male | 70 to 74 | 2015 | 14.46 | 10654 |
| Male | 70 to 74 | 2016 | 14.26 | 10785 |
| Male | 70 to 74 | 2017 | 14.15 | 11243 |
| Male | 70 to 74 | 2018 | 14.21 | 11833 |
| Male | 70 to 74 | 2019 | 14.09 | 12456 |
| Male | 70 to 74 | 2020 | 14.19 | 13071 |
| Male | 70 to 74 | 2021 | 14.32 | 13805 |
| Male | 70 to 74 | 2022 | 14.00 | 13853 |
| Male | 70 to 74 | 2023 | 13.93 | 14302 |
| Male | 70 to 74 | 2024 | 13.86 | 14735 |
| Male | 70 to 74 | 2025 | 13.86 | 15210 |
| Male | 70 to 74 | 2026 | 13.86 | 15652 |
| Male | 70 to 74 | 2027 | 13.85 | 16050 |
| Male | 70 to 74 | 2028 | 13.85 | 16350 |
| Male | 70 to 74 | 2029 | 13.84 | 16559 |
| Male | 70 to 74 | 2030 | 13.85 | 16769 |
| Male | 70 to 74 | 2031 | 13.86 | 17035 |
| Male | 70 to 74 | 2032 | 13.87 | 17423 |
| Male | 70 to 74 | 2033 | 13.88 | 17999 |
| Male | 70 to 74 | 2034 | 13.88 | 18725 |
| Male | 70 to 74 | 2035 | 13.92 | 19554 |
| Male | 70 to 74 | 2036 | 13.95 | 20366 |
| Male | 70 to 74 | 2037 | 13.99 | 21078 |
| Male | 70 to 74 | 2038 | 14.03 | 21678 |
| Male | 70 to 74 | 2039 | 14.06 | 22214 |
| Male | 70 to 74 | 2040 | 14.20 | 22865 |
| Male | 70 to 74 | 2041 | 14.35 | 23470 |
| Male | 70 to 74 | 2042 | 14.49 | 24033 |
| Male | 70 to 74 | 2043 | 14.63 | 24528 |
| Male | 70 to 74 | 2044 | 14.78 | 24953 |
| Male | 70 to 74 | 2045 | 14.92 | 25349 |
| Male | 70 to 74 | 2046 | 15.06 | 25759 |
| Male | 75 to 79 | 1992 | 42.13 | 10770 |
| Male | 75 to 79 | 1993 | 41.65 | 10705 |
| Male | 75 to 79 | 1994 | 40.30 | 10491 |
| Male | 75 to 79 | 1995 | 40.06 | 10735 |
| Male | 75 to 79 | 1996 | 39.84 | 11112 |
| Male | 75 to 79 | 1997 | 39.51 | 11526 |
| Male | 75 to 79 | 1998 | 39.52 | 12057 |
| Male | 75 to 79 | 1999 | 39.57 | 12621 |
| Male | 75 to 79 | 2000 | 38.20 | 12702 |
| Male | 75 to 79 | 2001 | 36.49 | 12628 |
| Male | 75 to 79 | 2002 | 35.53 | 12812 |
| Male | 75 to 79 | 2003 | 34.56 | 12996 |
| Male | 75 to 79 | 2004 | 33.16 | 12968 |
| Male | 75 to 79 | 2005 | 32.24 | 13052 |
| Male | 75 to 79 | 2006 | 31.06 | 12986 |
| Male | 75 to 79 | 2007 | 29.99 | 12879 |
| Male | 75 to 79 | 2008 | 29.02 | 12759 |
| Male | 75 to 79 | 2009 | 27.77 | 12499 |
| Male | 75 to 79 | 2010 | 26.84 | 12380 |
| Male | 75 to 79 | 2011 | 25.66 | 12158 |
| Male | 75 to 79 | 2012 | 24.95 | 12166 |
| Male | 75 to 79 | 2013 | 24.50 | 12283 |
| Male | 75 to 79 | 2014 | 24.24 | 12468 |
| Male | 75 to 79 | 2015 | 24.11 | 12713 |
| Male | 75 to 79 | 2016 | 23.91 | 12914 |
| Male | 75 to 79 | 2017 | 23.43 | 12921 |
| Male | 75 to 79 | 2018 | 23.06 | 13006 |
| Male | 75 to 79 | 2019 | 22.83 | 13160 |
| Male | 75 to 79 | 2020 | 22.51 | 13193 |
| Male | 75 to 79 | 2021 | 22.24 | 13297 |
| Male | 75 to 79 | 2022 | 22.16 | 13976 |
| Male | 75 to 79 | 2023 | 21.94 | 14458 |
| Male | 75 to 79 | 2024 | 21.73 | 15059 |
| Male | 75 to 79 | 2025 | 21.68 | 15821 |
| Male | 75 to 79 | 2026 | 21.62 | 16572 |
| Male | 75 to 79 | 2027 | 21.57 | 17254 |
| Male | 75 to 79 | 2028 | 21.52 | 17886 |
| Male | 75 to 79 | 2029 | 21.47 | 18508 |
| Male | 75 to 79 | 2030 | 21.49 | 19166 |
| Male | 75 to 79 | 2031 | 21.51 | 19783 |
| Male | 75 to 79 | 2032 | 21.53 | 20342 |
| Male | 75 to 79 | 2033 | 21.55 | 20779 |
| Male | 75 to 79 | 2034 | 21.57 | 21111 |
| Male | 75 to 79 | 2035 | 21.61 | 21450 |
| Male | 75 to 79 | 2036 | 21.64 | 21869 |
| Male | 75 to 79 | 2037 | 21.68 | 22448 |
| Male | 75 to 79 | 2038 | 21.72 | 23272 |
| Male | 75 to 79 | 2039 | 21.75 | 24294 |
| Male | 75 to 79 | 2040 | 21.81 | 25414 |
| Male | 75 to 79 | 2041 | 21.86 | 26504 |
| Male | 75 to 79 | 2042 | 21.91 | 27458 |
| Male | 75 to 79 | 2043 | 21.96 | 28268 |
| Male | 75 to 79 | 2044 | 22.01 | 29002 |
| Male | 75 to 79 | 2045 | 22.06 | 29665 |
| Male | 75 to 79 | 2046 | 22.11 | 30259 |
| Male | 80 to 84 | 1992 | 60.34 | 8512 |
| Male | 80 to 84 | 1993 | 61.03 | 8857 |
| Male | 80 to 84 | 1994 | 60.85 | 9049 |
| Male | 80 to 84 | 1995 | 61.12 | 9254 |
| Male | 80 to 84 | 1996 | 60.73 | 9292 |
| Male | 80 to 84 | 1997 | 59.22 | 9141 |
| Male | 80 to 84 | 1998 | 57.49 | 8970 |
| Male | 80 to 84 | 1999 | 56.22 | 8939 |
| Male | 80 to 84 | 2000 | 54.69 | 9028 |
| Male | 80 to 84 | 2001 | 53.47 | 9257 |
| Male | 80 to 84 | 2002 | 53.11 | 9672 |
| Male | 80 to 84 | 2003 | 53.34 | 10200 |
| Male | 80 to 84 | 2004 | 52.83 | 10597 |
| Male | 80 to 84 | 2005 | 51.23 | 10757 |
| Male | 80 to 84 | 2006 | 49.25 | 10817 |
| Male | 80 to 84 | 2007 | 47.75 | 10999 |
| Male | 80 to 84 | 2008 | 46.21 | 11169 |
| Male | 80 to 84 | 2009 | 44.67 | 11293 |
| Male | 80 to 84 | 2010 | 43.50 | 11448 |
| Male | 80 to 84 | 2011 | 41.98 | 11474 |
| Male | 80 to 84 | 2012 | 40.79 | 11516 |
| Male | 80 to 84 | 2013 | 39.66 | 11527 |
| Male | 80 to 84 | 2014 | 38.66 | 11559 |
| Male | 80 to 84 | 2015 | 37.77 | 11636 |
| Male | 80 to 84 | 2016 | 37.06 | 11797 |
| Male | 80 to 84 | 2017 | 36.26 | 11951 |
| Male | 80 to 84 | 2018 | 35.79 | 12195 |
| Male | 80 to 84 | 2019 | 35.38 | 12437 |
| Male | 80 to 84 | 2020 | 34.78 | 12536 |
| Male | 80 to 84 | 2021 | 34.72 | 12727 |
| Male | 80 to 84 | 2022 | 33.91 | 13083 |
| Male | 80 to 84 | 2023 | 33.42 | 13251 |
| Male | 80 to 84 | 2024 | 32.94 | 13410 |
| Male | 80 to 84 | 2025 | 32.70 | 13706 |
| Male | 80 to 84 | 2026 | 32.47 | 14068 |
| Male | 80 to 84 | 2027 | 32.23 | 14517 |
| Male | 80 to 84 | 2028 | 32.00 | 15113 |
| Male | 80 to 84 | 2029 | 31.76 | 15843 |
| Male | 80 to 84 | 2030 | 31.72 | 16725 |
| Male | 80 to 84 | 2031 | 31.69 | 17593 |
| Male | 80 to 84 | 2032 | 31.65 | 18381 |
| Male | 80 to 84 | 2033 | 31.62 | 19119 |
| Male | 80 to 84 | 2034 | 31.58 | 19859 |
| Male | 80 to 84 | 2035 | 31.64 | 20641 |
| Male | 80 to 84 | 2036 | 31.71 | 21379 |
| Male | 80 to 84 | 2037 | 31.77 | 22049 |
| Male | 80 to 84 | 2038 | 31.83 | 22596 |
| Male | 80 to 84 | 2039 | 31.89 | 23049 |
| Male | 80 to 84 | 2040 | 31.94 | 23499 |
| Male | 80 to 84 | 2041 | 31.99 | 24043 |
| Male | 80 to 84 | 2042 | 32.05 | 24760 |
| Male | 80 to 84 | 2043 | 32.10 | 25743 |
| Male | 80 to 84 | 2044 | 32.15 | 26942 |
| Male | 80 to 84 | 2045 | 32.20 | 28222 |
| Male | 80 to 84 | 2046 | 32.25 | 29459 |
| Male | 85 to 89 | 1992 | 80.94 | 4517 |
| Male | 85 to 89 | 1993 | 82.40 | 4787 |
| Male | 85 to 89 | 1994 | 82.19 | 4951 |
| Male | 85 to 89 | 1995 | 82.97 | 5171 |
| Male | 85 to 89 | 1996 | 84.11 | 5419 |
| Male | 85 to 89 | 1997 | 84.43 | 5620 |
| Male | 85 to 89 | 1998 | 84.77 | 5830 |
| Male | 85 to 89 | 1999 | 85.88 | 6088 |
| Male | 85 to 89 | 2000 | 85.45 | 6212 |
| Male | 85 to 89 | 2001 | 83.56 | 6185 |
| Male | 85 to 89 | 2002 | 82.31 | 6189 |
| Male | 85 to 89 | 2003 | 79.88 | 6104 |
| Male | 85 to 89 | 2004 | 77.09 | 6033 |
| Male | 85 to 89 | 2005 | 75.38 | 6173 |
| Male | 85 to 89 | 2006 | 74.52 | 6464 |
| Male | 85 to 89 | 2007 | 73.89 | 6815 |
| Male | 85 to 89 | 2008 | 72.88 | 7124 |
| Male | 85 to 89 | 2009 | 71.54 | 7404 |
| Male | 85 to 89 | 2010 | 70.38 | 7693 |
| Male | 85 to 89 | 2011 | 68.51 | 7871 |
| Male | 85 to 89 | 2012 | 66.83 | 8112 |
| Male | 85 to 89 | 2013 | 65.44 | 8390 |
| Male | 85 to 89 | 2014 | 64.44 | 8668 |
| Male | 85 to 89 | 2015 | 63.81 | 8959 |
| Male | 85 to 89 | 2016 | 63.23 | 9263 |
| Male | 85 to 89 | 2017 | 62.04 | 9430 |
| Male | 85 to 89 | 2018 | 60.52 | 9524 |
| Male | 85 to 89 | 2019 | 59.24 | 9670 |
| Male | 85 to 89 | 2020 | 58.49 | 9856 |
| Male | 85 to 89 | 2021 | 57.94 | 9997 |
| Male | 85 to 89 | 2022 | 55.74 | 10521 |
| Male | 85 to 89 | 2023 | 54.47 | 10608 |
| Male | 85 to 89 | 2024 | 53.19 | 10722 |
| Male | 85 to 89 | 2025 | 52.60 | 10994 |
| Male | 85 to 89 | 2026 | 52.01 | 11269 |
| Male | 85 to 89 | 2027 | 51.42 | 11529 |
| Male | 85 to 89 | 2028 | 50.83 | 11768 |
| Male | 85 to 89 | 2029 | 50.24 | 12018 |
| Male | 85 to 89 | 2030 | 49.95 | 12384 |
| Male | 85 to 89 | 2031 | 49.67 | 12813 |
| Male | 85 to 89 | 2032 | 49.39 | 13317 |
| Male | 85 to 89 | 2033 | 49.11 | 13966 |
| Male | 85 to 89 | 2034 | 48.83 | 14754 |
| Male | 85 to 89 | 2035 | 48.83 | 15678 |
| Male | 85 to 89 | 2036 | 48.83 | 16582 |
| Male | 85 to 89 | 2037 | 48.82 | 17397 |
| Male | 85 to 89 | 2038 | 48.82 | 18170 |
| Male | 85 to 89 | 2039 | 48.82 | 18960 |
| Male | 85 to 89 | 2040 | 48.91 | 19773 |
| Male | 85 to 89 | 2041 | 49.00 | 20546 |
| Male | 85 to 89 | 2042 | 49.09 | 21254 |
| Male | 85 to 89 | 2043 | 49.18 | 21854 |
| Male | 85 to 89 | 2044 | 49.26 | 22387 |
| Male | 85 to 89 | 2045 | 49.35 | 22939 |
| Male | 85 to 89 | 2046 | 49.44 | 23587 |
| Male | 90 to 94 | 1992 | 106.76 | 1517 |
| Male | 90 to 94 | 1993 | 106.68 | 1619 |
| Male | 90 to 94 | 1994 | 105.72 | 1714 |
| Male | 90 to 94 | 1995 | 105.92 | 1829 |
| Male | 90 to 94 | 1996 | 106.26 | 1939 |
| Male | 90 to 94 | 1997 | 105.78 | 2038 |
| Male | 90 to 94 | 1998 | 106.55 | 2148 |
| Male | 90 to 94 | 1999 | 107.49 | 2258 |
| Male | 90 to 94 | 2000 | 108.14 | 2361 |
| Male | 90 to 94 | 2001 | 108.19 | 2448 |
| Male | 90 to 94 | 2002 | 108.17 | 2537 |
| Male | 90 to 94 | 2003 | 109.00 | 2648 |
| Male | 90 to 94 | 2004 | 108.39 | 2724 |
| Male | 90 to 94 | 2005 | 108.23 | 2812 |
| Male | 90 to 94 | 2006 | 106.72 | 2858 |
| Male | 90 to 94 | 2007 | 103.77 | 2863 |
| Male | 90 to 94 | 2008 | 99.00 | 2820 |
| Male | 90 to 94 | 2009 | 94.75 | 2814 |
| Male | 90 to 94 | 2010 | 94.79 | 2998 |
| Male | 90 to 94 | 2011 | 94.10 | 3190 |
| Male | 90 to 94 | 2012 | 93.11 | 3387 |
| Male | 90 to 94 | 2013 | 93.19 | 3612 |
| Male | 90 to 94 | 2014 | 93.82 | 3849 |
| Male | 90 to 94 | 2015 | 94.06 | 4071 |
| Male | 90 to 94 | 2016 | 93.85 | 4286 |
| Male | 90 to 94 | 2017 | 93.04 | 4506 |
| Male | 90 to 94 | 2018 | 91.40 | 4701 |
| Male | 90 to 94 | 2019 | 90.02 | 4900 |
| Male | 90 to 94 | 2020 | 89.43 | 5078 |
| Male | 90 to 94 | 2021 | 89.29 | 5204 |
| Male | 90 to 94 | 2022 | 83.68 | 5570 |
| Male | 90 to 94 | 2023 | 81.40 | 5630 |
| Male | 90 to 94 | 2024 | 79.11 | 5746 |
| Male | 90 to 94 | 2025 | 77.84 | 5945 |
| Male | 90 to 94 | 2026 | 76.57 | 6121 |
| Male | 90 to 94 | 2027 | 75.29 | 6252 |
| Male | 90 to 94 | 2028 | 74.02 | 6384 |
| Male | 90 to 94 | 2029 | 72.75 | 6556 |
| Male | 90 to 94 | 2030 | 72.06 | 6797 |
| Male | 90 to 94 | 2031 | 71.36 | 7037 |
| Male | 90 to 94 | 2032 | 70.67 | 7257 |
| Male | 90 to 94 | 2033 | 69.97 | 7466 |
| Male | 90 to 94 | 2034 | 69.28 | 7694 |
| Male | 90 to 94 | 2035 | 68.98 | 7999 |
| Male | 90 to 94 | 2036 | 68.68 | 8349 |
| Male | 90 to 94 | 2037 | 68.38 | 8748 |
| Male | 90 to 94 | 2038 | 68.08 | 9254 |
| Male | 90 to 94 | 2039 | 67.78 | 9868 |
| Male | 90 to 94 | 2040 | 67.78 | 10562 |
| Male | 90 to 94 | 2041 | 67.78 | 11234 |
| Male | 90 to 94 | 2042 | 67.77 | 11834 |
| Male | 90 to 94 | 2043 | 67.77 | 12408 |
| Male | 90 to 94 | 2044 | 67.77 | 13011 |
| Male | 90 to 94 | 2045 | 67.77 | 13612 |
| Male | 90 to 94 | 2046 | 67.77 | 14186 |
| Female | 50 to 54 | 1992 | 0.81 | 864 |
| Female | 50 to 54 | 1993 | 0.82 | 883 |
| Female | 50 to 54 | 1994 | 0.83 | 898 |
| Female | 50 to 54 | 1995 | 0.81 | 876 |
| Female | 50 to 54 | 1996 | 0.79 | 866 |
| Female | 50 to 54 | 1997 | 0.77 | 875 |
| Female | 50 to 54 | 1998 | 0.76 | 897 |
| Female | 50 to 54 | 1999 | 0.78 | 965 |
| Female | 50 to 54 | 2000 | 0.80 | 1031 |
| Female | 50 to 54 | 2001 | 0.78 | 1065 |
| Female | 50 to 54 | 2002 | 0.79 | 1111 |
| Female | 50 to 54 | 2003 | 0.78 | 1135 |
| Female | 50 to 54 | 2004 | 0.76 | 1142 |
| Female | 50 to 54 | 2005 | 0.74 | 1165 |
| Female | 50 to 54 | 2006 | 0.73 | 1175 |
| Female | 50 to 54 | 2007 | 0.73 | 1202 |
| Female | 50 to 54 | 2008 | 0.73 | 1209 |
| Female | 50 to 54 | 2009 | 0.72 | 1208 |
| Female | 50 to 54 | 2010 | 0.70 | 1201 |
| Female | 50 to 54 | 2011 | 0.70 | 1219 |
| Female | 50 to 54 | 2012 | 0.70 | 1247 |
| Female | 50 to 54 | 2013 | 0.69 | 1280 |
| Female | 50 to 54 | 2014 | 0.68 | 1299 |
| Female | 50 to 54 | 2015 | 0.67 | 1330 |
| Female | 50 to 54 | 2016 | 0.66 | 1359 |
| Female | 50 to 54 | 2017 | 0.64 | 1345 |
| Female | 50 to 54 | 2018 | 0.63 | 1347 |
| Female | 50 to 54 | 2019 | 0.62 | 1346 |
| Female | 50 to 54 | 2020 | 0.61 | 1353 |
| Female | 50 to 54 | 2021 | 0.62 | 1384 |
| Female | 50 to 54 | 2022 | 0.64 | 1469 |
| Female | 50 to 54 | 2023 | 0.65 | 1495 |
| Female | 50 to 54 | 2024 | 0.65 | 1518 |
| Female | 50 to 54 | 2025 | 0.66 | 1536 |
| Female | 50 to 54 | 2026 | 0.66 | 1555 |
| Female | 50 to 54 | 2027 | 0.66 | 1577 |
| Female | 50 to 54 | 2028 | 0.67 | 1600 |
| Female | 50 to 54 | 2029 | 0.67 | 1625 |
| Female | 50 to 54 | 2030 | 0.67 | 1645 |
| Female | 50 to 54 | 2031 | 0.68 | 1669 |
| Female | 50 to 54 | 2032 | 0.68 | 1699 |
| Female | 50 to 54 | 2033 | 0.68 | 1736 |
| Female | 50 to 54 | 2034 | 0.68 | 1781 |
| Female | 50 to 54 | 2035 | 0.68 | 1824 |
| Female | 50 to 54 | 2036 | 0.68 | 1865 |
| Female | 50 to 54 | 2037 | 0.68 | 1899 |
| Female | 50 to 54 | 2038 | 0.68 | 1927 |
| Female | 50 to 54 | 2039 | 0.68 | 1953 |
| Female | 50 to 54 | 2040 | 0.68 | 1984 |
| Female | 50 to 54 | 2041 | 0.69 | 2009 |
| Female | 50 to 54 | 2042 | 0.69 | 2026 |
| Female | 50 to 54 | 2043 | 0.70 | 2031 |
| Female | 50 to 54 | 2044 | 0.70 | 2028 |
| Female | 50 to 54 | 2045 | 0.70 | 2020 |
| Female | 50 to 54 | 2046 | 0.71 | 2015 |
| Female | 55 to 59 | 1992 | 1.33 | 1270 |
| Female | 55 to 59 | 1993 | 1.39 | 1350 |
| Female | 55 to 59 | 1994 | 1.45 | 1434 |
| Female | 55 to 59 | 1995 | 1.44 | 1443 |
| Female | 55 to 59 | 1996 | 1.42 | 1435 |
| Female | 55 to 59 | 1997 | 1.40 | 1421 |
| Female | 55 to 59 | 1998 | 1.38 | 1401 |
| Female | 55 to 59 | 1999 | 1.37 | 1406 |
| Female | 55 to 59 | 2000 | 1.35 | 1386 |
| Female | 55 to 59 | 2001 | 1.31 | 1366 |
| Female | 55 to 59 | 2002 | 1.30 | 1410 |
| Female | 55 to 59 | 2003 | 1.29 | 1450 |
| Female | 55 to 59 | 2004 | 1.26 | 1506 |
| Female | 55 to 59 | 2005 | 1.28 | 1600 |
| Female | 55 to 59 | 2006 | 1.28 | 1683 |
| Female | 55 to 59 | 2007 | 1.27 | 1739 |
| Female | 55 to 59 | 2008 | 1.24 | 1760 |
| Female | 55 to 59 | 2009 | 1.20 | 1755 |
| Female | 55 to 59 | 2010 | 1.16 | 1776 |
| Female | 55 to 59 | 2011 | 1.16 | 1820 |
| Female | 55 to 59 | 2012 | 1.16 | 1858 |
| Female | 55 to 59 | 2013 | 1.17 | 1906 |
| Female | 55 to 59 | 2014 | 1.18 | 1940 |
| Female | 55 to 59 | 2015 | 1.19 | 1980 |
| Female | 55 to 59 | 2016 | 1.19 | 2021 |
| Female | 55 to 59 | 2017 | 1.18 | 2048 |
| Female | 55 to 59 | 2018 | 1.17 | 2110 |
| Female | 55 to 59 | 2019 | 1.17 | 2190 |
| Female | 55 to 59 | 2020 | 1.15 | 2248 |
| Female | 55 to 59 | 2021 | 1.14 | 2288 |
| Female | 55 to 59 | 2022 | 1.13 | 2335 |
| Female | 55 to 59 | 2023 | 1.12 | 2363 |
| Female | 55 to 59 | 2024 | 1.10 | 2381 |
| Female | 55 to 59 | 2025 | 1.11 | 2422 |
| Female | 55 to 59 | 2026 | 1.11 | 2460 |
| Female | 55 to 59 | 2027 | 1.12 | 2495 |
| Female | 55 to 59 | 2028 | 1.12 | 2526 |
| Female | 55 to 59 | 2029 | 1.12 | 2551 |
| Female | 55 to 59 | 2030 | 1.13 | 2580 |
| Female | 55 to 59 | 2031 | 1.14 | 2609 |
| Female | 55 to 59 | 2032 | 1.14 | 2644 |
| Female | 55 to 59 | 2033 | 1.15 | 2682 |
| Female | 55 to 59 | 2034 | 1.15 | 2720 |
| Female | 55 to 59 | 2035 | 1.16 | 2754 |
| Female | 55 to 59 | 2036 | 1.16 | 2794 |
| Female | 55 to 59 | 2037 | 1.16 | 2842 |
| Female | 55 to 59 | 2038 | 1.17 | 2905 |
| Female | 55 to 59 | 2039 | 1.17 | 2980 |
| Female | 55 to 59 | 2040 | 1.17 | 3052 |
| Female | 55 to 59 | 2041 | 1.17 | 3122 |
| Female | 55 to 59 | 2042 | 1.17 | 3180 |
| Female | 55 to 59 | 2043 | 1.17 | 3228 |
| Female | 55 to 59 | 2044 | 1.17 | 3271 |
| Female | 55 to 59 | 2045 | 1.17 | 3306 |
| Female | 55 to 59 | 2046 | 1.17 | 3330 |
| Female | 60 to 64 | 1992 | 2.43 | 2053 |
| Female | 60 to 64 | 1993 | 2.46 | 2105 |
| Female | 60 to 64 | 1994 | 2.51 | 2164 |
| Female | 60 to 64 | 1995 | 2.46 | 2152 |
| Female | 60 to 64 | 1996 | 2.40 | 2128 |
| Female | 60 to 64 | 1997 | 2.35 | 2124 |
| Female | 60 to 64 | 1998 | 2.31 | 2136 |
| Female | 60 to 64 | 1999 | 2.36 | 2217 |
| Female | 60 to 64 | 2000 | 2.36 | 2248 |
| Female | 60 to 64 | 2001 | 2.29 | 2210 |
| Female | 60 to 64 | 2002 | 2.28 | 2213 |
| Female | 60 to 64 | 2003 | 2.24 | 2188 |
| Female | 60 to 64 | 2004 | 2.22 | 2178 |
| Female | 60 to 64 | 2005 | 2.21 | 2182 |
| Female | 60 to 64 | 2006 | 2.15 | 2164 |
| Female | 60 to 64 | 2007 | 2.12 | 2225 |
| Female | 60 to 64 | 2008 | 2.08 | 2286 |
| Female | 60 to 64 | 2009 | 2.06 | 2392 |
| Female | 60 to 64 | 2010 | 2.07 | 2522 |
| Female | 60 to 64 | 2011 | 2.09 | 2683 |
| Female | 60 to 64 | 2012 | 2.03 | 2707 |
| Female | 60 to 64 | 2013 | 1.99 | 2759 |
| Female | 60 to 64 | 2014 | 1.99 | 2844 |
| Female | 60 to 64 | 2015 | 1.95 | 2904 |
| Female | 60 to 64 | 2016 | 1.95 | 2987 |
| Female | 60 to 64 | 2017 | 1.95 | 3047 |
| Female | 60 to 64 | 2018 | 1.97 | 3129 |
| Female | 60 to 64 | 2019 | 2.01 | 3219 |
| Female | 60 to 64 | 2020 | 2.02 | 3264 |
| Female | 60 to 64 | 2021 | 2.02 | 3323 |
| Female | 60 to 64 | 2022 | 1.98 | 3344 |
| Female | 60 to 64 | 2023 | 1.97 | 3432 |
| Female | 60 to 64 | 2024 | 1.97 | 3547 |
| Female | 60 to 64 | 2025 | 1.95 | 3652 |
| Female | 60 to 64 | 2026 | 1.93 | 3752 |
| Female | 60 to 64 | 2027 | 1.92 | 3830 |
| Female | 60 to 64 | 2028 | 1.90 | 3882 |
| Female | 60 to 64 | 2029 | 1.89 | 3917 |
| Female | 60 to 64 | 2030 | 1.89 | 3985 |
| Female | 60 to 64 | 2031 | 1.90 | 4046 |
| Female | 60 to 64 | 2032 | 1.90 | 4103 |
| Female | 60 to 64 | 2033 | 1.91 | 4152 |
| Female | 60 to 64 | 2034 | 1.91 | 4192 |
| Female | 60 to 64 | 2035 | 1.92 | 4236 |
| Female | 60 to 64 | 2036 | 1.93 | 4283 |
| Female | 60 to 64 | 2037 | 1.94 | 4337 |
| Female | 60 to 64 | 2038 | 1.95 | 4397 |
| Female | 60 to 64 | 2039 | 1.96 | 4458 |
| Female | 60 to 64 | 2040 | 1.96 | 4513 |
| Female | 60 to 64 | 2041 | 1.97 | 4578 |
| Female | 60 to 64 | 2042 | 1.97 | 4658 |
| Female | 60 to 64 | 2043 | 1.98 | 4761 |
| Female | 60 to 64 | 2044 | 1.98 | 4885 |
| Female | 60 to 64 | 2045 | 1.98 | 5017 |
| Female | 60 to 64 | 2046 | 1.99 | 5144 |
| Female | 65 to 69 | 1992 | 4.74 | 3314 |
| Female | 65 to 69 | 1993 | 4.82 | 3448 |
| Female | 65 to 69 | 1994 | 4.90 | 3586 |
| Female | 65 to 69 | 1995 | 4.86 | 3620 |
| Female | 65 to 69 | 1996 | 4.78 | 3620 |
| Female | 65 to 69 | 1997 | 4.66 | 3574 |
| Female | 65 to 69 | 1998 | 4.59 | 3565 |
| Female | 65 to 69 | 1999 | 4.59 | 3607 |
| Female | 65 to 69 | 2000 | 4.49 | 3572 |
| Female | 65 to 69 | 2001 | 4.28 | 3469 |
| Female | 65 to 69 | 2002 | 4.16 | 3453 |
| Female | 65 to 69 | 2003 | 4.10 | 3473 |
| Female | 65 to 69 | 2004 | 4.08 | 3526 |
| Female | 65 to 69 | 2005 | 4.10 | 3606 |
| Female | 65 to 69 | 2006 | 4.03 | 3590 |
| Female | 65 to 69 | 2007 | 3.99 | 3585 |
| Female | 65 to 69 | 2008 | 3.90 | 3534 |
| Female | 65 to 69 | 2009 | 3.85 | 3520 |
| Female | 65 to 69 | 2010 | 3.78 | 3489 |
| Female | 65 to 69 | 2011 | 3.72 | 3503 |
| Female | 65 to 69 | 2012 | 3.65 | 3592 |
| Female | 65 to 69 | 2013 | 3.61 | 3719 |
| Female | 65 to 69 | 2014 | 3.60 | 3935 |
| Female | 65 to 69 | 2015 | 3.67 | 4201 |
| Female | 65 to 69 | 2016 | 3.73 | 4508 |
| Female | 65 to 69 | 2017 | 3.69 | 4637 |
| Female | 65 to 69 | 2018 | 3.66 | 4786 |
| Female | 65 to 69 | 2019 | 3.62 | 4894 |
| Female | 65 to 69 | 2020 | 3.54 | 4972 |
| Female | 65 to 69 | 2021 | 3.51 | 5058 |
| Female | 65 to 69 | 2022 | 3.62 | 5332 |
| Female | 65 to 69 | 2023 | 3.62 | 5443 |
| Female | 65 to 69 | 2024 | 3.63 | 5522 |
| Female | 65 to 69 | 2025 | 3.62 | 5570 |
| Female | 65 to 69 | 2026 | 3.61 | 5633 |
| Female | 65 to 69 | 2027 | 3.60 | 5732 |
| Female | 65 to 69 | 2028 | 3.59 | 5888 |
| Female | 65 to 69 | 2029 | 3.58 | 6090 |
| Female | 65 to 69 | 2030 | 3.55 | 6282 |
| Female | 65 to 69 | 2031 | 3.52 | 6462 |
| Female | 65 to 69 | 2032 | 3.50 | 6605 |
| Female | 65 to 69 | 2033 | 3.47 | 6704 |
| Female | 65 to 69 | 2034 | 3.45 | 6773 |
| Female | 65 to 69 | 2035 | 3.45 | 6890 |
| Female | 65 to 69 | 2036 | 3.46 | 6995 |
| Female | 65 to 69 | 2037 | 3.47 | 7092 |
| Female | 65 to 69 | 2038 | 3.48 | 7177 |
| Female | 65 to 69 | 2039 | 3.49 | 7245 |
| Female | 65 to 69 | 2040 | 3.50 | 7320 |
| Female | 65 to 69 | 2041 | 3.52 | 7398 |
| Female | 65 to 69 | 2042 | 3.53 | 7491 |
| Female | 65 to 69 | 2043 | 3.55 | 7592 |
| Female | 65 to 69 | 2044 | 3.57 | 7697 |
| Female | 65 to 69 | 2045 | 3.58 | 7811 |
| Female | 65 to 69 | 2046 | 3.60 | 7945 |
| Female | 70 to 74 | 1992 | 8.87 | 4502 |
| Female | 70 to 74 | 1993 | 9.05 | 4800 |
| Female | 70 to 74 | 1994 | 9.20 | 5067 |
| Female | 70 to 74 | 1995 | 9.17 | 5222 |
| Female | 70 to 74 | 1996 | 9.08 | 5317 |
| Female | 70 to 74 | 1997 | 8.89 | 5351 |
| Female | 70 to 74 | 1998 | 8.80 | 5430 |
| Female | 70 to 74 | 1999 | 8.94 | 5651 |
| Female | 70 to 74 | 2000 | 8.66 | 5587 |
| Female | 70 to 74 | 2001 | 8.15 | 5359 |
| Female | 70 to 74 | 2002 | 7.97 | 5323 |
| Female | 70 to 74 | 2003 | 7.84 | 5311 |
| Female | 70 to 74 | 2004 | 7.70 | 5285 |
| Female | 70 to 74 | 2005 | 7.55 | 5276 |
| Female | 70 to 74 | 2006 | 7.28 | 5194 |
| Female | 70 to 74 | 2007 | 7.14 | 5232 |
| Female | 70 to 74 | 2008 | 6.99 | 5246 |
| Female | 70 to 74 | 2009 | 6.90 | 5297 |
| Female | 70 to 74 | 2010 | 6.83 | 5350 |
| Female | 70 to 74 | 2011 | 6.77 | 5390 |
| Female | 70 to 74 | 2012 | 6.71 | 5403 |
| Female | 70 to 74 | 2013 | 6.65 | 5407 |
| Female | 70 to 74 | 2014 | 6.62 | 5451 |
| Female | 70 to 74 | 2015 | 6.58 | 5477 |
| Female | 70 to 74 | 2016 | 6.56 | 5580 |
| Female | 70 to 74 | 2017 | 6.46 | 5752 |
| Female | 70 to 74 | 2018 | 6.45 | 6021 |
| Female | 70 to 74 | 2019 | 6.39 | 6333 |
| Female | 70 to 74 | 2020 | 6.42 | 6676 |
| Female | 70 to 74 | 2021 | 6.59 | 7212 |
| Female | 70 to 74 | 2022 | 6.44 | 7331 |
| Female | 70 to 74 | 2023 | 6.43 | 7615 |
| Female | 70 to 74 | 2024 | 6.42 | 7886 |
| Female | 70 to 74 | 2025 | 6.42 | 8159 |
| Female | 70 to 74 | 2026 | 6.43 | 8413 |
| Female | 70 to 74 | 2027 | 6.43 | 8644 |
| Female | 70 to 74 | 2028 | 6.43 | 8824 |
| Female | 70 to 74 | 2029 | 6.44 | 8954 |
| Female | 70 to 74 | 2030 | 6.42 | 9043 |
| Female | 70 to 74 | 2031 | 6.41 | 9156 |
| Female | 70 to 74 | 2032 | 6.39 | 9328 |
| Female | 70 to 74 | 2033 | 6.37 | 9596 |
| Female | 70 to 74 | 2034 | 6.36 | 9939 |
| Female | 70 to 74 | 2035 | 6.32 | 10270 |
| Female | 70 to 74 | 2036 | 6.27 | 10583 |
| Female | 70 to 74 | 2037 | 6.23 | 10834 |
| Female | 70 to 74 | 2038 | 6.19 | 11012 |
| Female | 70 to 74 | 2039 | 6.15 | 11142 |
| Female | 70 to 74 | 2040 | 6.16 | 11339 |
| Female | 70 to 74 | 2041 | 6.17 | 11514 |
| Female | 70 to 74 | 2042 | 6.19 | 11678 |
| Female | 70 to 74 | 2043 | 6.20 | 11821 |
| Female | 70 to 74 | 2044 | 6.22 | 11935 |
| Female | 70 to 74 | 2045 | 6.23 | 12039 |
| Female | 70 to 74 | 2046 | 6.24 | 12149 |
| Female | 75 to 79 | 1992 | 16.35 | 5916 |
| Female | 75 to 79 | 1993 | 16.27 | 5852 |
| Female | 75 to 79 | 1994 | 16.29 | 5892 |
| Female | 75 to 79 | 1995 | 16.24 | 6011 |
| Female | 75 to 79 | 1996 | 16.20 | 6228 |
| Female | 75 to 79 | 1997 | 16.08 | 6460 |
| Female | 75 to 79 | 1998 | 16.06 | 6766 |
| Female | 75 to 79 | 1999 | 16.51 | 7254 |
| Female | 75 to 79 | 2000 | 16.19 | 7370 |
| Female | 75 to 79 | 2001 | 15.40 | 7232 |
| Female | 75 to 79 | 2002 | 15.00 | 7255 |
| Female | 75 to 79 | 2003 | 14.66 | 7293 |
| Female | 75 to 79 | 2004 | 14.33 | 7325 |
| Female | 75 to 79 | 2005 | 13.97 | 7317 |
| Female | 75 to 79 | 2006 | 13.58 | 7273 |
| Female | 75 to 79 | 2007 | 13.36 | 7297 |
| Female | 75 to 79 | 2008 | 13.04 | 7256 |
| Female | 75 to 79 | 2009 | 12.77 | 7237 |
| Female | 75 to 79 | 2010 | 12.51 | 7245 |
| Female | 75 to 79 | 2011 | 12.21 | 7253 |
| Female | 75 to 79 | 2012 | 11.97 | 7320 |
| Female | 75 to 79 | 2013 | 11.80 | 7423 |
| Female | 75 to 79 | 2014 | 11.83 | 7632 |
| Female | 75 to 79 | 2015 | 11.85 | 7829 |
| Female | 75 to 79 | 2016 | 11.90 | 8010 |
| Female | 75 to 79 | 2017 | 11.86 | 8093 |
| Female | 75 to 79 | 2018 | 11.76 | 8122 |
| Female | 75 to 79 | 2019 | 11.81 | 8269 |
| Female | 75 to 79 | 2020 | 11.56 | 8179 |
| Female | 75 to 79 | 2021 | 11.39 | 8211 |
| Female | 75 to 79 | 2022 | 11.54 | 8809 |
| Female | 75 to 79 | 2023 | 11.49 | 9181 |
| Female | 75 to 79 | 2024 | 11.45 | 9656 |
| Female | 75 to 79 | 2025 | 11.43 | 10199 |
| Female | 75 to 79 | 2026 | 11.40 | 10741 |
| Female | 75 to 79 | 2027 | 11.38 | 11232 |
| Female | 75 to 79 | 2028 | 11.36 | 11676 |
| Female | 75 to 79 | 2029 | 11.34 | 12103 |
| Female | 75 to 79 | 2030 | 11.35 | 12537 |
| Female | 75 to 79 | 2031 | 11.35 | 12942 |
| Female | 75 to 79 | 2032 | 11.36 | 13311 |
| Female | 75 to 79 | 2033 | 11.36 | 13601 |
| Female | 75 to 79 | 2034 | 11.37 | 13816 |
| Female | 75 to 79 | 2035 | 11.34 | 13977 |
| Female | 75 to 79 | 2036 | 11.32 | 14178 |
| Female | 75 to 79 | 2037 | 11.29 | 14472 |
| Female | 75 to 79 | 2038 | 11.27 | 14916 |
| Female | 75 to 79 | 2039 | 11.24 | 15480 |
| Female | 75 to 79 | 2040 | 11.17 | 16032 |
| Female | 75 to 79 | 2041 | 11.11 | 16552 |
| Female | 75 to 79 | 2042 | 11.04 | 16973 |
| Female | 75 to 79 | 2043 | 10.97 | 17280 |
| Female | 75 to 79 | 2044 | 10.91 | 17513 |
| Female | 75 to 79 | 2045 | 10.84 | 17692 |
| Female | 75 to 79 | 2046 | 10.77 | 17831 |
| Female | 80 to 84 | 1992 | 25.81 | 6040 |
| Female | 80 to 84 | 1993 | 26.04 | 6265 |
| Female | 80 to 84 | 1994 | 26.41 | 6490 |
| Female | 80 to 84 | 1995 | 26.81 | 6686 |
| Female | 80 to 84 | 1996 | 26.98 | 6736 |
| Female | 80 to 84 | 1997 | 26.78 | 6671 |
| Female | 80 to 84 | 1998 | 26.57 | 6602 |
| Female | 80 to 84 | 1999 | 26.92 | 6768 |
| Female | 80 to 84 | 2000 | 26.30 | 6821 |
| Female | 80 to 84 | 2001 | 25.16 | 6833 |
| Female | 80 to 84 | 2002 | 24.77 | 7076 |
| Female | 80 to 84 | 2003 | 24.86 | 7473 |
| Female | 80 to 84 | 2004 | 24.74 | 7784 |
| Female | 80 to 84 | 2005 | 24.16 | 7909 |
| Female | 80 to 84 | 2006 | 23.55 | 7990 |
| Female | 80 to 84 | 2007 | 23.02 | 8083 |
| Female | 80 to 84 | 2008 | 22.32 | 8099 |
| Female | 80 to 84 | 2009 | 21.99 | 8237 |
| Female | 80 to 84 | 2010 | 21.69 | 8355 |
| Female | 80 to 84 | 2011 | 21.34 | 8444 |
| Female | 80 to 84 | 2012 | 21.12 | 8545 |
| Female | 80 to 84 | 2013 | 20.79 | 8604 |
| Female | 80 to 84 | 2014 | 20.59 | 8712 |
| Female | 80 to 84 | 2015 | 20.34 | 8832 |
| Female | 80 to 84 | 2016 | 20.10 | 8994 |
| Female | 80 to 84 | 2017 | 19.80 | 9158 |
| Female | 80 to 84 | 2018 | 19.62 | 9363 |
| Female | 80 to 84 | 2019 | 19.63 | 9629 |
| Female | 80 to 84 | 2020 | 19.37 | 9723 |
| Female | 80 to 84 | 2021 | 19.30 | 9830 |
| Female | 80 to 84 | 2022 | 19.36 | 10213 |
| Female | 80 to 84 | 2023 | 19.30 | 10401 |
| Female | 80 to 84 | 2024 | 19.24 | 10569 |
| Female | 80 to 84 | 2025 | 19.17 | 10761 |
| Female | 80 to 84 | 2026 | 19.11 | 11022 |
| Female | 80 to 84 | 2027 | 19.04 | 11387 |
| Female | 80 to 84 | 2028 | 18.97 | 11910 |
| Female | 80 to 84 | 2029 | 18.90 | 12571 |
| Female | 80 to 84 | 2030 | 18.87 | 13315 |
| Female | 80 to 84 | 2031 | 18.83 | 14053 |
| Female | 80 to 84 | 2032 | 18.80 | 14717 |
| Female | 80 to 84 | 2033 | 18.77 | 15321 |
| Female | 80 to 84 | 2034 | 18.73 | 15911 |
| Female | 80 to 84 | 2035 | 18.74 | 16509 |
| Female | 80 to 84 | 2036 | 18.75 | 17069 |
| Female | 80 to 84 | 2037 | 18.76 | 17576 |
| Female | 80 to 84 | 2038 | 18.77 | 17981 |
| Female | 80 to 84 | 2039 | 18.78 | 18298 |
| Female | 80 to 84 | 2040 | 18.74 | 18559 |
| Female | 80 to 84 | 2041 | 18.70 | 18878 |
| Female | 80 to 84 | 2042 | 18.66 | 19323 |
| Female | 80 to 84 | 2043 | 18.62 | 19967 |
| Female | 80 to 84 | 2044 | 18.58 | 20772 |
| Female | 80 to 84 | 2045 | 18.54 | 21632 |
| Female | 80 to 84 | 2046 | 18.50 | 22449 |
| Female | 85 to 89 | 1992 | 40.66 | 4466 |
| Female | 85 to 89 | 1993 | 41.10 | 4685 |
| Female | 85 to 89 | 1994 | 41.68 | 4925 |
| Female | 85 to 89 | 1995 | 42.38 | 5167 |
| Female | 85 to 89 | 1996 | 42.55 | 5362 |
| Female | 85 to 89 | 1997 | 42.16 | 5479 |
| Female | 85 to 89 | 1998 | 42.62 | 5729 |
| Female | 85 to 89 | 1999 | 44.81 | 6195 |
| Female | 85 to 89 | 2000 | 44.94 | 6348 |
| Female | 85 to 89 | 2001 | 43.52 | 6198 |
| Female | 85 to 89 | 2002 | 42.56 | 6083 |
| Female | 85 to 89 | 2003 | 42.13 | 6042 |
| Female | 85 to 89 | 2004 | 41.25 | 6025 |
| Female | 85 to 89 | 2005 | 40.46 | 6153 |
| Female | 85 to 89 | 2006 | 39.84 | 6406 |
| Female | 85 to 89 | 2007 | 39.50 | 6741 |
| Female | 85 to 89 | 2008 | 39.00 | 7062 |
| Female | 85 to 89 | 2009 | 38.67 | 7383 |
| Female | 85 to 89 | 2010 | 38.22 | 7645 |
| Female | 85 to 89 | 2011 | 37.67 | 7845 |
| Female | 85 to 89 | 2012 | 37.23 | 8062 |
| Female | 85 to 89 | 2013 | 36.87 | 8288 |
| Female | 85 to 89 | 2014 | 37.03 | 8623 |
| Female | 85 to 89 | 2015 | 37.23 | 8948 |
| Female | 85 to 89 | 2016 | 37.48 | 9295 |
| Female | 85 to 89 | 2017 | 37.01 | 9429 |
| Female | 85 to 89 | 2018 | 36.31 | 9508 |
| Female | 85 to 89 | 2019 | 35.68 | 9617 |
| Female | 85 to 89 | 2020 | 34.39 | 9541 |
| Female | 85 to 89 | 2021 | 34.09 | 9705 |
| Female | 85 to 89 | 2022 | 34.35 | 10260 |
| Female | 85 to 89 | 2023 | 33.98 | 10420 |
| Female | 85 to 89 | 2024 | 33.61 | 10595 |
| Female | 85 to 89 | 2025 | 33.51 | 10870 |
| Female | 85 to 89 | 2026 | 33.41 | 11152 |
| Female | 85 to 89 | 2027 | 33.31 | 11429 |
| Female | 85 to 89 | 2028 | 33.21 | 11677 |
| Female | 85 to 89 | 2029 | 33.12 | 11920 |
| Female | 85 to 89 | 2030 | 33.01 | 12203 |
| Female | 85 to 89 | 2031 | 32.90 | 12571 |
| Female | 85 to 89 | 2032 | 32.79 | 13055 |
| Female | 85 to 89 | 2033 | 32.68 | 13724 |
| Female | 85 to 89 | 2034 | 32.58 | 14557 |
| Female | 85 to 89 | 2035 | 32.52 | 15481 |
| Female | 85 to 89 | 2036 | 32.47 | 16385 |
| Female | 85 to 89 | 2037 | 32.42 | 17189 |
| Female | 85 to 89 | 2038 | 32.36 | 17925 |
| Female | 85 to 89 | 2039 | 32.31 | 18658 |
| Female | 85 to 89 | 2040 | 32.32 | 19404 |
| Female | 85 to 89 | 2041 | 32.34 | 20105 |
| Female | 85 to 89 | 2042 | 32.35 | 20738 |
| Female | 85 to 89 | 2043 | 32.36 | 21257 |
| Female | 85 to 89 | 2044 | 32.37 | 21693 |
| Female | 85 to 89 | 2045 | 32.39 | 22132 |
| Female | 85 to 89 | 2046 | 32.40 | 22651 |
| Female | 90 to 94 | 1992 | 60.33 | 2065 |
| Female | 90 to 94 | 1993 | 60.61 | 2204 |
| Female | 90 to 94 | 1994 | 61.52 | 2370 |
| Female | 90 to 94 | 1995 | 61.29 | 2489 |
| Female | 90 to 94 | 1996 | 61.08 | 2600 |
| Female | 90 to 94 | 1997 | 60.56 | 2714 |
| Female | 90 to 94 | 1998 | 61.49 | 2884 |
| Female | 90 to 94 | 1999 | 63.67 | 3122 |
| Female | 90 to 94 | 2000 | 64.56 | 3297 |
| Female | 90 to 94 | 2001 | 63.64 | 3389 |
| Female | 90 to 94 | 2002 | 63.37 | 3501 |
| Female | 90 to 94 | 2003 | 63.23 | 3629 |
| Female | 90 to 94 | 2004 | 63.47 | 3770 |
| Female | 90 to 94 | 2005 | 63.39 | 3874 |
| Female | 90 to 94 | 2006 | 63.32 | 3939 |
| Female | 90 to 94 | 2007 | 63.30 | 3995 |
| Female | 90 to 94 | 2008 | 62.19 | 3991 |
| Female | 90 to 94 | 2009 | 60.49 | 4013 |
| Female | 90 to 94 | 2010 | 59.99 | 4225 |
| Female | 90 to 94 | 2011 | 59.89 | 4519 |
| Female | 90 to 94 | 2012 | 59.82 | 4836 |
| Female | 90 to 94 | 2013 | 60.20 | 5187 |
| Female | 90 to 94 | 2014 | 60.63 | 5521 |
| Female | 90 to 94 | 2015 | 60.59 | 5795 |
| Female | 90 to 94 | 2016 | 61.53 | 6153 |
| Female | 90 to 94 | 2017 | 61.18 | 6388 |
| Female | 90 to 94 | 2018 | 60.62 | 6608 |
| Female | 90 to 94 | 2019 | 59.89 | 6814 |
| Female | 90 to 94 | 2020 | 58.09 | 6833 |
| Female | 90 to 94 | 2021 | 57.91 | 6984 |
| Female | 90 to 94 | 2022 | 57.48 | 7315 |
| Female | 90 to 94 | 2023 | 56.82 | 7441 |
| Female | 90 to 94 | 2024 | 56.15 | 7612 |
| Female | 90 to 94 | 2025 | 55.68 | 7825 |
| Female | 90 to 94 | 2026 | 55.20 | 8030 |
| Female | 90 to 94 | 2027 | 54.72 | 8204 |
| Female | 90 to 94 | 2028 | 54.25 | 8379 |
| Female | 90 to 94 | 2029 | 53.77 | 8584 |
| Female | 90 to 94 | 2030 | 53.62 | 8861 |
| Female | 90 to 94 | 2031 | 53.48 | 9142 |
| Female | 90 to 94 | 2032 | 53.33 | 9412 |
| Female | 90 to 94 | 2033 | 53.18 | 9663 |
| Female | 90 to 94 | 2034 | 53.04 | 9925 |
| Female | 90 to 94 | 2035 | 52.88 | 10232 |
| Female | 90 to 94 | 2036 | 52.72 | 10614 |
| Female | 90 to 94 | 2037 | 52.56 | 11091 |
| Female | 90 to 94 | 2038 | 52.40 | 11732 |
| Female | 90 to 94 | 2039 | 52.24 | 12518 |
| Female | 90 to 94 | 2040 | 52.16 | 13375 |
| Female | 90 to 94 | 2041 | 52.08 | 14201 |
| Female | 90 to 94 | 2042 | 52.00 | 14923 |
| Female | 90 to 94 | 2043 | 51.93 | 15589 |
| Female | 90 to 94 | 2044 | 51.85 | 16268 |
| Female | 90 to 94 | 2045 | 51.77 | 16933 |
| Female | 90 to 94 | 2046 | 51.69 | 17558 |
| Both | 50 to 54 | 1992 | 1.48 | 3202 |
| Both | 50 to 54 | 1993 | 1.53 | 3325 |
| Both | 50 to 54 | 1994 | 1.54 | 3357 |
| Both | 50 to 54 | 1995 | 1.52 | 3316 |
| Both | 50 to 54 | 1996 | 1.47 | 3264 |
| Both | 50 to 54 | 1997 | 1.44 | 3298 |
| Both | 50 to 54 | 1998 | 1.42 | 3389 |
| Both | 50 to 54 | 1999 | 1.44 | 3617 |
| Both | 50 to 54 | 2000 | 1.49 | 3881 |
| Both | 50 to 54 | 2001 | 1.48 | 4066 |
| Both | 50 to 54 | 2002 | 1.49 | 4219 |
| Both | 50 to 54 | 2003 | 1.47 | 4331 |
| Both | 50 to 54 | 2004 | 1.44 | 4353 |
| Both | 50 to 54 | 2005 | 1.43 | 4506 |
| Both | 50 to 54 | 2006 | 1.40 | 4524 |
| Both | 50 to 54 | 2007 | 1.39 | 4593 |
| Both | 50 to 54 | 2008 | 1.39 | 4662 |
| Both | 50 to 54 | 2009 | 1.37 | 4641 |
| Both | 50 to 54 | 2010 | 1.36 | 4652 |
| Both | 50 to 54 | 2011 | 1.35 | 4697 |
| Both | 50 to 54 | 2012 | 1.33 | 4754 |
| Both | 50 to 54 | 2013 | 1.31 | 4838 |
| Both | 50 to 54 | 2014 | 1.29 | 4968 |
| Both | 50 to 54 | 2015 | 1.27 | 5075 |
| Both | 50 to 54 | 2016 | 1.27 | 5224 |
| Both | 50 to 54 | 2017 | 1.24 | 5213 |
| Both | 50 to 54 | 2018 | 1.23 | 5274 |
| Both | 50 to 54 | 2019 | 1.23 | 5344 |
| Both | 50 to 54 | 2020 | 1.21 | 5324 |
| Both | 50 to 54 | 2021 | 1.21 | 5405 |
| Both | 50 to 54 | 2022 | 1.27 | 5845 |
| Both | 50 to 54 | 2023 | 1.29 | 5968 |
| Both | 50 to 54 | 2024 | 1.30 | 6075 |
| Both | 50 to 54 | 2025 | 1.32 | 6181 |
| Both | 50 to 54 | 2026 | 1.34 | 6290 |
| Both | 50 to 54 | 2027 | 1.35 | 6412 |
| Both | 50 to 54 | 2028 | 1.37 | 6542 |
| Both | 50 to 54 | 2029 | 1.39 | 6675 |
| Both | 50 to 54 | 2030 | 1.40 | 6800 |
| Both | 50 to 54 | 2031 | 1.41 | 6940 |
| Both | 50 to 54 | 2032 | 1.42 | 7103 |
| Both | 50 to 54 | 2033 | 1.44 | 7304 |
| Both | 50 to 54 | 2034 | 1.45 | 7537 |
| Both | 50 to 54 | 2035 | 1.46 | 7778 |
| Both | 50 to 54 | 2036 | 1.47 | 8011 |
| Both | 50 to 54 | 2037 | 1.48 | 8217 |
| Both | 50 to 54 | 2038 | 1.50 | 8397 |
| Both | 50 to 54 | 2039 | 1.51 | 8562 |
| Both | 50 to 54 | 2040 | 1.52 | 8712 |
| Both | 50 to 54 | 2041 | 1.53 | 8840 |
| Both | 50 to 54 | 2042 | 1.54 | 8940 |
| Both | 50 to 54 | 2043 | 1.56 | 9000 |
| Both | 50 to 54 | 2044 | 1.57 | 9027 |
| Both | 50 to 54 | 2045 | 1.58 | 9042 |
| Both | 50 to 54 | 2046 | 1.60 | 9067 |
| Both | 55 to 59 | 1992 | 2.55 | 4882 |
| Both | 55 to 59 | 1993 | 2.67 | 5193 |
| Both | 55 to 59 | 1994 | 2.79 | 5494 |
| Both | 55 to 59 | 1995 | 2.82 | 5624 |
| Both | 55 to 59 | 1996 | 2.77 | 5583 |
| Both | 55 to 59 | 1997 | 2.70 | 5464 |
| Both | 55 to 59 | 1998 | 2.63 | 5347 |
| Both | 55 to 59 | 1999 | 2.62 | 5342 |
| Both | 55 to 59 | 2000 | 2.58 | 5285 |
| Both | 55 to 59 | 2001 | 2.51 | 5226 |
| Both | 55 to 59 | 2002 | 2.50 | 5407 |
| Both | 55 to 59 | 2003 | 2.47 | 5579 |
| Both | 55 to 59 | 2004 | 2.43 | 5794 |
| Both | 55 to 59 | 2005 | 2.50 | 6209 |
| Both | 55 to 59 | 2006 | 2.48 | 6486 |
| Both | 55 to 59 | 2007 | 2.45 | 6637 |
| Both | 55 to 59 | 2008 | 2.40 | 6757 |
| Both | 55 to 59 | 2009 | 2.32 | 6723 |
| Both | 55 to 59 | 2010 | 2.26 | 6827 |
| Both | 55 to 59 | 2011 | 2.23 | 6930 |
| Both | 55 to 59 | 2012 | 2.22 | 7020 |
| Both | 55 to 59 | 2013 | 2.21 | 7118 |
| Both | 55 to 59 | 2014 | 2.23 | 7272 |
| Both | 55 to 59 | 2015 | 2.25 | 7406 |
| Both | 55 to 59 | 2016 | 2.27 | 7613 |
| Both | 55 to 59 | 2017 | 2.24 | 7678 |
| Both | 55 to 59 | 2018 | 2.23 | 7928 |
| Both | 55 to 59 | 2019 | 2.19 | 8099 |
| Both | 55 to 59 | 2020 | 2.13 | 8174 |
| Both | 55 to 59 | 2021 | 2.09 | 8290 |
| Both | 55 to 59 | 2022 | 2.16 | 8819 |
| Both | 55 to 59 | 2023 | 2.15 | 8999 |
| Both | 55 to 59 | 2024 | 2.15 | 9146 |
| Both | 55 to 59 | 2025 | 2.17 | 9370 |
| Both | 55 to 59 | 2026 | 2.18 | 9576 |
| Both | 55 to 59 | 2027 | 2.20 | 9769 |
| Both | 55 to 59 | 2028 | 2.22 | 9937 |
| Both | 55 to 59 | 2029 | 2.24 | 10077 |
| Both | 55 to 59 | 2030 | 2.27 | 10254 |
| Both | 55 to 59 | 2031 | 2.29 | 10436 |
| Both | 55 to 59 | 2032 | 2.32 | 10640 |
| Both | 55 to 59 | 2033 | 2.35 | 10858 |
| Both | 55 to 59 | 2034 | 2.38 | 11080 |
| Both | 55 to 59 | 2035 | 2.40 | 11293 |
| Both | 55 to 59 | 2036 | 2.42 | 11532 |
| Both | 55 to 59 | 2037 | 2.45 | 11809 |
| Both | 55 to 59 | 2038 | 2.47 | 12150 |
| Both | 55 to 59 | 2039 | 2.49 | 12547 |
| Both | 55 to 59 | 2040 | 2.51 | 12941 |
| Both | 55 to 59 | 2041 | 2.53 | 13323 |
| Both | 55 to 59 | 2042 | 2.55 | 13659 |
| Both | 55 to 59 | 2043 | 2.56 | 13951 |
| Both | 55 to 59 | 2044 | 2.58 | 14219 |
| Both | 55 to 59 | 2045 | 2.60 | 14455 |
| Both | 55 to 59 | 2046 | 2.61 | 14652 |
| Both | 60 to 64 | 1992 | 4.92 | 8177 |
| Both | 60 to 64 | 1993 | 4.99 | 8392 |
| Both | 60 to 64 | 1994 | 5.00 | 8494 |
| Both | 60 to 64 | 1995 | 4.92 | 8459 |
| Both | 60 to 64 | 1996 | 4.77 | 8317 |
| Both | 60 to 64 | 1997 | 4.63 | 8223 |
| Both | 60 to 64 | 1998 | 4.56 | 8247 |
| Both | 60 to 64 | 1999 | 4.62 | 8478 |
| Both | 60 to 64 | 2000 | 4.60 | 8573 |
| Both | 60 to 64 | 2001 | 4.49 | 8455 |
| Both | 60 to 64 | 2002 | 4.45 | 8448 |
| Both | 60 to 64 | 2003 | 4.38 | 8376 |
| Both | 60 to 64 | 2004 | 4.26 | 8210 |
| Both | 60 to 64 | 2005 | 4.20 | 8155 |
| Both | 60 to 64 | 2006 | 4.05 | 8024 |
| Both | 60 to 64 | 2007 | 4.01 | 8283 |
| Both | 60 to 64 | 2008 | 3.96 | 8536 |
| Both | 60 to 64 | 2009 | 3.90 | 8894 |
| Both | 60 to 64 | 2010 | 3.93 | 9369 |
| Both | 60 to 64 | 2011 | 3.90 | 9813 |
| Both | 60 to 64 | 2012 | 3.80 | 9887 |
| Both | 60 to 64 | 2013 | 3.71 | 10034 |
| Both | 60 to 64 | 2014 | 3.67 | 10233 |
| Both | 60 to 64 | 2015 | 3.61 | 10493 |
| Both | 60 to 64 | 2016 | 3.63 | 10829 |
| Both | 60 to 64 | 2017 | 3.61 | 10981 |
| Both | 60 to 64 | 2018 | 3.66 | 11318 |
| Both | 60 to 64 | 2019 | 3.69 | 11536 |
| Both | 60 to 64 | 2020 | 3.66 | 11559 |
| Both | 60 to 64 | 2021 | 3.65 | 11669 |
| Both | 60 to 64 | 2022 | 3.63 | 11928 |
| Both | 60 to 64 | 2023 | 3.62 | 12253 |
| Both | 60 to 64 | 2024 | 3.61 | 12675 |
| Both | 60 to 64 | 2025 | 3.60 | 13136 |
| Both | 60 to 64 | 2026 | 3.60 | 13581 |
| Both | 60 to 64 | 2027 | 3.59 | 13959 |
| Both | 60 to 64 | 2028 | 3.58 | 14256 |
| Both | 60 to 64 | 2029 | 3.58 | 14500 |
| Both | 60 to 64 | 2030 | 3.61 | 14865 |
| Both | 60 to 64 | 2031 | 3.64 | 15200 |
| Both | 60 to 64 | 2032 | 3.68 | 15515 |
| Both | 60 to 64 | 2033 | 3.71 | 15790 |
| Both | 60 to 64 | 2034 | 3.74 | 16021 |
| Both | 60 to 64 | 2035 | 3.78 | 16304 |
| Both | 60 to 64 | 2036 | 3.83 | 16595 |
| Both | 60 to 64 | 2037 | 3.88 | 16921 |
| Both | 60 to 64 | 2038 | 3.92 | 17270 |
| Both | 60 to 64 | 2039 | 3.97 | 17625 |
| Both | 60 to 64 | 2040 | 4.00 | 17950 |
| Both | 60 to 64 | 2041 | 4.04 | 18318 |
| Both | 60 to 64 | 2042 | 4.07 | 18749 |
| Both | 60 to 64 | 2043 | 4.11 | 19281 |
| Both | 60 to 64 | 2044 | 4.14 | 19903 |
| Both | 60 to 64 | 2045 | 4.17 | 20563 |
| Both | 60 to 64 | 2046 | 4.21 | 21205 |
| Both | 65 to 69 | 1992 | 9.31 | 12220 |
| Both | 65 to 69 | 1993 | 9.47 | 12802 |
| Both | 65 to 69 | 1994 | 9.53 | 13230 |
| Both | 65 to 69 | 1995 | 9.49 | 13475 |
| Both | 65 to 69 | 1996 | 9.26 | 13411 |
| Both | 65 to 69 | 1997 | 8.99 | 13220 |
| Both | 65 to 69 | 1998 | 8.77 | 13046 |
| Both | 65 to 69 | 1999 | 8.66 | 13026 |
| Both | 65 to 69 | 2000 | 8.38 | 12779 |
| Both | 65 to 69 | 2001 | 7.98 | 12396 |
| Both | 65 to 69 | 2002 | 7.82 | 12405 |
| Both | 65 to 69 | 2003 | 7.67 | 12414 |
| Both | 65 to 69 | 2004 | 7.52 | 12403 |
| Both | 65 to 69 | 2005 | 7.46 | 12527 |
| Both | 65 to 69 | 2006 | 7.29 | 12410 |
| Both | 65 to 69 | 2007 | 7.19 | 12375 |
| Both | 65 to 69 | 2008 | 7.01 | 12199 |
| Both | 65 to 69 | 2009 | 6.87 | 12088 |
| Both | 65 to 69 | 2010 | 6.72 | 11963 |
| Both | 65 to 69 | 2011 | 6.51 | 11847 |
| Both | 65 to 69 | 2012 | 6.33 | 12048 |
| Both | 65 to 69 | 2013 | 6.25 | 12442 |
| Both | 65 to 69 | 2014 | 6.21 | 13122 |
| Both | 65 to 69 | 2015 | 6.32 | 13968 |
| Both | 65 to 69 | 2016 | 6.42 | 14950 |
| Both | 65 to 69 | 2017 | 6.37 | 15395 |
| Both | 65 to 69 | 2018 | 6.32 | 15889 |
| Both | 65 to 69 | 2019 | 6.25 | 16184 |
| Both | 65 to 69 | 2020 | 6.07 | 16363 |
| Both | 65 to 69 | 2021 | 5.98 | 16484 |
| Both | 65 to 69 | 2022 | 6.17 | 17360 |
| Both | 65 to 69 | 2023 | 6.16 | 17665 |
| Both | 65 to 69 | 2024 | 6.15 | 17868 |
| Both | 65 to 69 | 2025 | 6.14 | 18036 |
| Both | 65 to 69 | 2026 | 6.13 | 18259 |
| Both | 65 to 69 | 2027 | 6.13 | 18608 |
| Both | 65 to 69 | 2028 | 6.12 | 19151 |
| Both | 65 to 69 | 2029 | 6.11 | 19848 |
| Both | 65 to 69 | 2030 | 6.10 | 20611 |
| Both | 65 to 69 | 2031 | 6.10 | 21348 |
| Both | 65 to 69 | 2032 | 6.09 | 21977 |
| Both | 65 to 69 | 2033 | 6.09 | 22479 |
| Both | 65 to 69 | 2034 | 6.08 | 22902 |
| Both | 65 to 69 | 2035 | 6.14 | 23490 |
| Both | 65 to 69 | 2036 | 6.20 | 24033 |
| Both | 65 to 69 | 2037 | 6.25 | 24541 |
| Both | 65 to 69 | 2038 | 6.30 | 24987 |
| Both | 65 to 69 | 2039 | 6.36 | 25364 |
| Both | 65 to 69 | 2040 | 6.43 | 25787 |
| Both | 65 to 69 | 2041 | 6.50 | 26224 |
| Both | 65 to 69 | 2042 | 6.57 | 26716 |
| Both | 65 to 69 | 2043 | 6.64 | 27245 |
| Both | 65 to 69 | 2044 | 6.71 | 27783 |
| Both | 65 to 69 | 2045 | 6.78 | 28361 |
| Both | 65 to 69 | 2046 | 6.84 | 29010 |
| Both | 70 to 74 | 1992 | 16.09 | 14703 |
| Both | 70 to 74 | 1993 | 16.45 | 15693 |
| Both | 70 to 74 | 1994 | 16.64 | 16508 |
| Both | 70 to 74 | 1995 | 16.55 | 16994 |
| Both | 70 to 74 | 1996 | 16.21 | 17174 |
| Both | 70 to 74 | 1997 | 15.75 | 17216 |
| Both | 70 to 74 | 1998 | 15.46 | 17437 |
| Both | 70 to 74 | 1999 | 15.44 | 17927 |
| Both | 70 to 74 | 2000 | 14.93 | 17782 |
| Both | 70 to 74 | 2001 | 14.19 | 17288 |
| Both | 70 to 74 | 2002 | 13.88 | 17212 |
| Both | 70 to 74 | 2003 | 13.50 | 17001 |
| Both | 70 to 74 | 2004 | 13.04 | 16665 |
| Both | 70 to 74 | 2005 | 12.68 | 16487 |
| Both | 70 to 74 | 2006 | 12.12 | 16108 |
| Both | 70 to 74 | 2007 | 11.85 | 16152 |
| Both | 70 to 74 | 2008 | 11.60 | 16193 |
| Both | 70 to 74 | 2009 | 11.36 | 16219 |
| Both | 70 to 74 | 2010 | 11.21 | 16336 |
| Both | 70 to 74 | 2011 | 10.95 | 16233 |
| Both | 70 to 74 | 2012 | 10.75 | 16161 |
| Both | 70 to 74 | 2013 | 10.52 | 16034 |
| Both | 70 to 74 | 2014 | 10.40 | 16083 |
| Both | 70 to 74 | 2015 | 10.28 | 16131 |
| Both | 70 to 74 | 2016 | 10.19 | 16365 |
| Both | 70 to 74 | 2017 | 10.09 | 16995 |
| Both | 70 to 74 | 2018 | 10.11 | 17854 |
| Both | 70 to 74 | 2019 | 10.02 | 18789 |
| Both | 70 to 74 | 2020 | 10.07 | 19747 |
| Both | 70 to 74 | 2021 | 10.21 | 21017 |
| Both | 70 to 74 | 2022 | 9.95 | 21184 |
| Both | 70 to 74 | 2023 | 9.91 | 21917 |
| Both | 70 to 74 | 2024 | 9.87 | 22621 |
| Both | 70 to 74 | 2025 | 9.87 | 23369 |
| Both | 70 to 74 | 2026 | 9.87 | 24065 |
| Both | 70 to 74 | 2027 | 9.87 | 24694 |
| Both | 70 to 74 | 2028 | 9.86 | 25174 |
| Both | 70 to 74 | 2029 | 9.86 | 25513 |
| Both | 70 to 74 | 2030 | 9.86 | 25811 |
| Both | 70 to 74 | 2031 | 9.85 | 26190 |
| Both | 70 to 74 | 2032 | 9.85 | 26752 |
| Both | 70 to 74 | 2033 | 9.85 | 27595 |
| Both | 70 to 74 | 2034 | 9.84 | 28665 |
| Both | 70 to 74 | 2035 | 9.84 | 29825 |
| Both | 70 to 74 | 2036 | 9.84 | 30948 |
| Both | 70 to 74 | 2037 | 9.83 | 31912 |
| Both | 70 to 74 | 2038 | 9.83 | 32691 |
| Both | 70 to 74 | 2039 | 9.83 | 33356 |
| Both | 70 to 74 | 2040 | 9.91 | 34204 |
| Both | 70 to 74 | 2041 | 9.99 | 34984 |
| Both | 70 to 74 | 2042 | 10.07 | 35712 |
| Both | 70 to 74 | 2043 | 10.15 | 36349 |
| Both | 70 to 74 | 2044 | 10.22 | 36888 |
| Both | 70 to 74 | 2045 | 10.30 | 37388 |
| Both | 70 to 74 | 2046 | 10.37 | 37907 |
| Both | 75 to 79 | 1992 | 27.02 | 16686 |
| Both | 75 to 79 | 1993 | 26.85 | 16557 |
| Both | 75 to 79 | 1994 | 26.34 | 16383 |
| Both | 75 to 79 | 1995 | 26.25 | 16746 |
| Both | 75 to 79 | 1996 | 26.14 | 17341 |
| Both | 75 to 79 | 1997 | 25.93 | 17986 |
| Both | 75 to 79 | 1998 | 25.91 | 18823 |
| Both | 75 to 79 | 1999 | 26.21 | 19875 |
| Both | 75 to 79 | 2000 | 25.48 | 20072 |
| Both | 75 to 79 | 2001 | 24.35 | 19860 |
| Both | 75 to 79 | 2002 | 23.77 | 20067 |
| Both | 75 to 79 | 2003 | 23.23 | 20289 |
| Both | 75 to 79 | 2004 | 22.49 | 20294 |
| Both | 75 to 79 | 2005 | 21.94 | 20368 |
| Both | 75 to 79 | 2006 | 21.24 | 20259 |
| Both | 75 to 79 | 2007 | 20.68 | 20176 |
| Both | 75 to 79 | 2008 | 20.10 | 20015 |
| Both | 75 to 79 | 2009 | 19.41 | 19736 |
| Both | 75 to 79 | 2010 | 18.87 | 19624 |
| Both | 75 to 79 | 2011 | 18.18 | 19411 |
| Both | 75 to 79 | 2012 | 17.72 | 19486 |
| Both | 75 to 79 | 2013 | 17.43 | 19705 |
| Both | 75 to 79 | 2014 | 17.33 | 20100 |
| Both | 75 to 79 | 2015 | 17.29 | 20542 |
| Both | 75 to 79 | 2016 | 17.25 | 20924 |
| Both | 75 to 79 | 2017 | 17.03 | 21014 |
| Both | 75 to 79 | 2018 | 16.84 | 21128 |
| Both | 75 to 79 | 2019 | 16.78 | 21429 |
| Both | 75 to 79 | 2020 | 16.52 | 21372 |
| Both | 75 to 79 | 2021 | 16.31 | 21508 |
| Both | 75 to 79 | 2022 | 16.34 | 22785 |
| Both | 75 to 79 | 2023 | 16.22 | 23639 |
| Both | 75 to 79 | 2024 | 16.08 | 24715 |
| Both | 75 to 79 | 2025 | 16.04 | 26021 |
| Both | 75 to 79 | 2026 | 15.99 | 27313 |
| Both | 75 to 79 | 2027 | 15.94 | 28486 |
| Both | 75 to 79 | 2028 | 15.90 | 29562 |
| Both | 75 to 79 | 2029 | 15.87 | 30611 |
| Both | 75 to 79 | 2030 | 15.88 | 31703 |
| Both | 75 to 79 | 2031 | 15.89 | 32726 |
| Both | 75 to 79 | 2032 | 15.90 | 33653 |
| Both | 75 to 79 | 2033 | 15.91 | 34380 |
| Both | 75 to 79 | 2034 | 15.92 | 34927 |
| Both | 75 to 79 | 2035 | 15.92 | 35427 |
| Both | 75 to 79 | 2036 | 15.93 | 36047 |
| Both | 75 to 79 | 2037 | 15.93 | 36920 |
| Both | 75 to 79 | 2038 | 15.94 | 38188 |
| Both | 75 to 79 | 2039 | 15.95 | 39774 |
| Both | 75 to 79 | 2040 | 15.94 | 41445 |
| Both | 75 to 79 | 2041 | 15.93 | 43056 |
| Both | 75 to 79 | 2042 | 15.92 | 44431 |
| Both | 75 to 79 | 2043 | 15.91 | 45548 |
| Both | 75 to 79 | 2044 | 15.91 | 46516 |
| Both | 75 to 79 | 2045 | 15.91 | 47356 |
| Both | 75 to 79 | 2046 | 15.90 | 48090 |
| Both | 80 to 84 | 1992 | 38.80 | 14552 |
| Both | 80 to 84 | 1993 | 39.21 | 15122 |
| Both | 80 to 84 | 1994 | 39.40 | 15540 |
| Both | 80 to 84 | 1995 | 39.77 | 15940 |
| Both | 80 to 84 | 1996 | 39.81 | 16028 |
| Both | 80 to 84 | 1997 | 39.19 | 15812 |
| Both | 80 to 84 | 1998 | 38.49 | 15572 |
| Both | 80 to 84 | 1999 | 38.27 | 15708 |
| Both | 80 to 84 | 2000 | 37.34 | 15849 |
| Both | 80 to 84 | 2001 | 36.18 | 16090 |
| Both | 80 to 84 | 2002 | 35.81 | 16748 |
| Both | 80 to 84 | 2003 | 35.93 | 17673 |
| Both | 80 to 84 | 2004 | 35.68 | 18381 |
| Both | 80 to 84 | 2005 | 34.74 | 18666 |
| Both | 80 to 84 | 2006 | 33.65 | 18806 |
| Both | 80 to 84 | 2007 | 32.81 | 19082 |
| Both | 80 to 84 | 2008 | 31.87 | 19268 |
| Both | 80 to 84 | 2009 | 31.13 | 19531 |
| Both | 80 to 84 | 2010 | 30.55 | 19803 |
| Both | 80 to 84 | 2011 | 29.78 | 19917 |
| Both | 80 to 84 | 2012 | 29.21 | 20061 |
| Both | 80 to 84 | 2013 | 28.58 | 20131 |
| Both | 80 to 84 | 2014 | 28.07 | 20271 |
| Both | 80 to 84 | 2015 | 27.57 | 20468 |
| Both | 80 to 84 | 2016 | 27.15 | 20791 |
| Both | 80 to 84 | 2017 | 26.65 | 21109 |
| Both | 80 to 84 | 2018 | 26.36 | 21558 |
| Both | 80 to 84 | 2019 | 26.21 | 22066 |
| Both | 80 to 84 | 2020 | 25.81 | 22258 |
| Both | 80 to 84 | 2021 | 25.75 | 22557 |
| Both | 80 to 84 | 2022 | 25.51 | 23296 |
| Both | 80 to 84 | 2023 | 25.29 | 23652 |
| Both | 80 to 84 | 2024 | 25.07 | 23979 |
| Both | 80 to 84 | 2025 | 24.96 | 24466 |
| Both | 80 to 84 | 2026 | 24.84 | 25090 |
| Both | 80 to 84 | 2027 | 24.70 | 25904 |
| Both | 80 to 84 | 2028 | 24.56 | 27023 |
| Both | 80 to 84 | 2029 | 24.41 | 28413 |
| Both | 80 to 84 | 2030 | 24.36 | 30041 |
| Both | 80 to 84 | 2031 | 24.32 | 31646 |
| Both | 80 to 84 | 2032 | 24.27 | 33097 |
| Both | 80 to 84 | 2033 | 24.24 | 34440 |
| Both | 80 to 84 | 2034 | 24.20 | 35770 |
| Both | 80 to 84 | 2035 | 24.23 | 37150 |
| Both | 80 to 84 | 2036 | 24.26 | 38448 |
| Both | 80 to 84 | 2037 | 24.29 | 39625 |
| Both | 80 to 84 | 2038 | 24.33 | 40576 |
| Both | 80 to 84 | 2039 | 24.36 | 41347 |
| Both | 80 to 84 | 2040 | 24.36 | 42058 |
| Both | 80 to 84 | 2041 | 24.37 | 42922 |
| Both | 80 to 84 | 2042 | 24.38 | 44083 |
| Both | 80 to 84 | 2043 | 24.38 | 45710 |
| Both | 80 to 84 | 2044 | 24.39 | 47714 |
| Both | 80 to 84 | 2045 | 24.40 | 49855 |
| Both | 80 to 84 | 2046 | 24.40 | 51908 |
| Both | 85 to 89 | 1992 | 54.23 | 8983 |
| Both | 85 to 89 | 1993 | 55.04 | 9472 |
| Both | 85 to 89 | 1994 | 55.36 | 9877 |
| Both | 85 to 89 | 1995 | 56.11 | 10338 |
| Both | 85 to 89 | 1996 | 56.61 | 10781 |
| Both | 85 to 89 | 1997 | 56.48 | 11099 |
| Both | 85 to 89 | 1998 | 56.89 | 11560 |
| Both | 85 to 89 | 1999 | 58.73 | 12283 |
| Both | 85 to 89 | 2000 | 58.71 | 12560 |
| Both | 85 to 89 | 2001 | 57.21 | 12383 |
| Both | 85 to 89 | 2002 | 56.26 | 12271 |
| Both | 85 to 89 | 2003 | 55.25 | 12146 |
| Both | 85 to 89 | 2004 | 53.75 | 12058 |
| Both | 85 to 89 | 2005 | 52.68 | 12326 |
| Both | 85 to 89 | 2006 | 52.00 | 12870 |
| Both | 85 to 89 | 2007 | 51.56 | 13556 |
| Both | 85 to 89 | 2008 | 50.88 | 14187 |
| Both | 85 to 89 | 2009 | 50.22 | 14787 |
| Both | 85 to 89 | 2010 | 49.58 | 15338 |
| Both | 85 to 89 | 2011 | 48.63 | 15716 |
| Both | 85 to 89 | 2012 | 47.86 | 16174 |
| Both | 85 to 89 | 2013 | 47.25 | 16677 |
| Both | 85 to 89 | 2014 | 47.06 | 17291 |
| Both | 85 to 89 | 2015 | 47.03 | 17907 |
| Both | 85 to 89 | 2016 | 47.04 | 18558 |
| Both | 85 to 89 | 2017 | 46.37 | 18858 |
| Both | 85 to 89 | 2018 | 45.40 | 19032 |
| Both | 85 to 89 | 2019 | 44.57 | 19288 |
| Both | 85 to 89 | 2020 | 43.50 | 19397 |
| Both | 85 to 89 | 2021 | 43.09 | 19701 |
| Both | 85 to 89 | 2022 | 42.63 | 20781 |
| Both | 85 to 89 | 2023 | 41.94 | 21028 |
| Both | 85 to 89 | 2024 | 41.25 | 21318 |
| Both | 85 to 89 | 2025 | 40.99 | 21864 |
| Both | 85 to 89 | 2026 | 40.73 | 22421 |
| Both | 85 to 89 | 2027 | 40.47 | 22958 |
| Both | 85 to 89 | 2028 | 40.21 | 23445 |
| Both | 85 to 89 | 2029 | 39.95 | 23938 |
| Both | 85 to 89 | 2030 | 39.81 | 24587 |
| Both | 85 to 89 | 2031 | 39.66 | 25384 |
| Both | 85 to 89 | 2032 | 39.49 | 26373 |
| Both | 85 to 89 | 2033 | 39.32 | 27690 |
| Both | 85 to 89 | 2034 | 39.13 | 29311 |
| Both | 85 to 89 | 2035 | 39.09 | 31160 |
| Both | 85 to 89 | 2036 | 39.05 | 32967 |
| Both | 85 to 89 | 2037 | 39.01 | 34587 |
| Both | 85 to 89 | 2038 | 38.98 | 36095 |
| Both | 85 to 89 | 2039 | 38.95 | 37618 |
| Both | 85 to 89 | 2040 | 39.00 | 39177 |
| Both | 85 to 89 | 2041 | 39.05 | 40651 |
| Both | 85 to 89 | 2042 | 39.10 | 41992 |
| Both | 85 to 89 | 2043 | 39.15 | 43112 |
| Both | 85 to 89 | 2044 | 39.20 | 44080 |
| Both | 85 to 89 | 2045 | 39.25 | 45071 |
| Both | 85 to 89 | 2046 | 39.31 | 46239 |
| Both | 90 to 94 | 1992 | 73.95 | 3582 |
| Both | 90 to 94 | 1993 | 74.17 | 3823 |
| Both | 90 to 94 | 1994 | 74.61 | 4084 |
| Both | 90 to 94 | 1995 | 74.61 | 4318 |
| Both | 90 to 94 | 1996 | 74.64 | 4539 |
| Both | 90 to 94 | 1997 | 74.16 | 4752 |
| Both | 90 to 94 | 1998 | 75.04 | 5032 |
| Both | 90 to 94 | 1999 | 76.81 | 5380 |
| Both | 90 to 94 | 2000 | 77.61 | 5658 |
| Both | 90 to 94 | 2001 | 76.92 | 5837 |
| Both | 90 to 94 | 2002 | 76.72 | 6038 |
| Both | 90 to 94 | 2003 | 76.84 | 6276 |
| Both | 90 to 94 | 2004 | 76.83 | 6495 |
| Both | 90 to 94 | 2005 | 76.77 | 6686 |
| Both | 90 to 94 | 2006 | 76.38 | 6796 |
| Both | 90 to 94 | 2007 | 75.61 | 6859 |
| Both | 90 to 94 | 2008 | 73.50 | 6811 |
| Both | 90 to 94 | 2009 | 71.08 | 6827 |
| Both | 90 to 94 | 2010 | 70.78 | 7223 |
| Both | 90 to 94 | 2011 | 70.49 | 7709 |
| Both | 90 to 94 | 2012 | 70.15 | 8223 |
| Both | 90 to 94 | 2013 | 70.43 | 8799 |
| Both | 90 to 94 | 2014 | 70.94 | 9370 |
| Both | 90 to 94 | 2015 | 71.02 | 9865 |
| Both | 90 to 94 | 2016 | 71.66 | 10440 |
| Both | 90 to 94 | 2017 | 71.27 | 10894 |
| Both | 90 to 94 | 2018 | 70.49 | 11309 |
| Both | 90 to 94 | 2019 | 69.64 | 11714 |
| Both | 90 to 94 | 2020 | 68.29 | 11911 |
| Both | 90 to 94 | 2021 | 68.13 | 12188 |
| Both | 90 to 94 | 2022 | 66.48 | 12885 |
| Both | 90 to 94 | 2023 | 65.31 | 13072 |
| Both | 90 to 94 | 2024 | 64.16 | 13358 |
| Both | 90 to 94 | 2025 | 63.48 | 13770 |
| Both | 90 to 94 | 2026 | 62.78 | 14151 |
| Both | 90 to 94 | 2027 | 62.06 | 14456 |
| Both | 90 to 94 | 2028 | 61.33 | 14763 |
| Both | 90 to 94 | 2029 | 60.62 | 15140 |
| Both | 90 to 94 | 2030 | 60.32 | 15658 |
| Both | 90 to 94 | 2031 | 60.02 | 16178 |
| Both | 90 to 94 | 2032 | 59.71 | 16669 |
| Both | 90 to 94 | 2033 | 59.40 | 17129 |
| Both | 90 to 94 | 2034 | 59.09 | 17619 |
| Both | 90 to 94 | 2035 | 58.91 | 18232 |
| Both | 90 to 94 | 2036 | 58.73 | 18963 |
| Both | 90 to 94 | 2037 | 58.53 | 19839 |
| Both | 90 to 94 | 2038 | 58.32 | 20986 |
| Both | 90 to 94 | 2039 | 58.11 | 22386 |
| Both | 90 to 94 | 2040 | 58.06 | 23937 |
| Both | 90 to 94 | 2041 | 58.01 | 25435 |
| Both | 90 to 94 | 2042 | 57.97 | 26757 |
| Both | 90 to 94 | 2043 | 57.93 | 27997 |
| Both | 90 to 94 | 2044 | 57.89 | 29279 |
| Both | 90 to 94 | 2045 | 57.86 | 30546 |
| Both | 90 to 94 | 2046 | 57.82 | 31744 |
